# Intraperitoneal injection of IFN-γ restores microglial autophagy, promotes amyloid-β clearance and improves cognition in APP/PS1 mice

**DOI:** 10.1038/s41419-020-2644-4

**Published:** 2020-06-08

**Authors:** Zitian He, Yunjie Yang, Zhiwei Xing, Zejie Zuo, Rui Wang, Huaiyu Gu, Fangfang Qi, Zhibin Yao

**Affiliations:** 1 Department of Anatomy and Neurobiology, Zhongshan School of Medicine, Sun Yat-sen University, #74, Zhongshan No. 2 Road, 510080 Guangzhou, China; 2 Guangdong Province Key Laboratory of Brain Function and Disease, Zhongshan School of Medicine, Sun Yat-sen University, #74, Zhongshan No. 2 Road, 510080 Guangzhou, China; 3 Teaching and Research Bureau of Surgery, Sun Yat-Sen Memorial Hospital, Sun Yat-Sen University, 510120 Guangzhou, Guangdong China

**Keywords:** Cognitive neuroscience, Neuroimmunology

## Abstract

Autophagy is a major self-degradative process that maintains cellular homeostasis and function in mammalian cells. Autophagic dysfunction occurs in the early pathogenesis of Alzheimer’s disease (AD) and directly regulates amyloid-β (Aβ) metabolism. Although it has been proven that the cytokine IFN-γ enhances autophagy in macrophage cell lines, whether the signaling cascade is implicated in Aβ degradation in AD mouse models remains to be elucidated. Here, we found that 9 days of the intraperitoneal administration of IFN-γ significantly increased the LC3II/I ratio and decreased the level of p62 in APP/PS1 mice, an AD mouse model. In vitro, IFN-γ protected BV2 cells from Aβ toxicity by upregulating the expressions of Atg7 and Atg5 and the LC3II/I ratio, whereas these protective effects were ablated by interference with Atg5 expression. Moreover, IFN-γ enhanced autophagic flux, probably through suppressing the AKT/mTOR pathway both in vivo and in vitro. Importantly, using intravital two-photon microscopy and fluorescence staining, we found that microglia interacted with exogenous IFN-γ and Aβ, and surrounded Aβ in APP/PS1;CX3CR1-GFP^+/−^ mice. In addition, IFN-γ treatment decreased the Aβ plaque load in the cortex and hippocampus and rescued cognitive deficits in APP/PS1 mice. Our data suggest a possible mechanism by which the peripheral injection of IFN-γ restores microglial autophagy to induce the phagocytosis of cerebral Aβ, which represents a potential therapeutic approach for the use of exogenous IFN-γ in AD.

## Background

Alzheimer’s disease (AD), which is the most common type of dementia in older people, is characterized by the abnormal accumulation of amyloid-β (Aβ) and intracellular neurofibrillary tangles (NFTs) in the brain, which results in progressive synaptic dysfunction and cognitive deficits^1–4^. An imbalance between protein production and degradation contributes to the accumulation of the proteinaceous inclusions characteristic of neurodegenerative disorders, including Aβ and tau in Alzheimer’s disease^5^. Increasing evidence has shown that increased protein turnover may promote disease progression in AD^6–8^. Therefore, the alteration of immunoproteostasis could be a valuable therapeutic strategy to alleviate AD pathology.

The autophagy–lysosome system (hereafter autophagy) and the ubiquitin–proteasome system represent two major independent intracellular degradation pathways for proteinaceous inclusions resulting from sporadic biosynthetic errors or misfolding. Autophagy is an important cellular pathway for the degradation and clearance of damaged organelles and denatured and aggregated peptides^9^. It is a highly conserved homeostatic process by which cytoplasmic macromolecules, excess or damaged organelles, and some pathogens are delivered to lysosomes for degradation^10^. Previous studies have shown that autophagic dysfunction in the brain causes neurodegeneration in mice and that defects in autophagosome formation and autophagosome-lysosome fusion occur early during AD pathogenesis^11–13^.

The type II interferon (IFN) IFN-γ is a cytokine that is mainly secreted by activated T helper type 1 (Th1) lymphocytes and natural killer (NK) cells^14,15^. It is critical for cell autonomous innate immunity against bacteria, protozoa, viruses, and fungi^16,17^. It has been reported that AAV-induced murine IFN-γ expression in the neonatal brain of APP mice reduces Aβ accumulation through the synergistic effects of activated glia and complement expression that promote Aβ clearance. However, no behavioral or cognitive effects were observed after IFN-γ expression in the neonatal brain^18^. Moreover, PD-1 immune checkpoint blockade reduces pathology and improves memory in mouse models of Alzheimer’s disease by evoking an IFN-γ-dependent systemic immune response, which is followed by the recruitment of macrophages to the brain^19^. Notably, it has been reported that IFN-γ could elicit macrophage autophagy mediated by PI3K and p38 MAPK in vitro^20^.

Due to the complexity and long-term effects of gene modification, in this study, the intraperitoneal injection of IFN-γ was used in 8-month-old APP/PS1 mice to explore the therapeutic effects and underlying mechanisms of IFN-γ. We found that the intraperitoneal injection of IFN-γ rescued cognitive impairment in APP/PS1 mice, reduced Aβ deposition, and initiated autophagy via the AKT/mTOR pathway; these effects were blocked in microglia BV2 cells via interference with the Atg5 gene.

## Results

### IFN-γ treatment increased autophagy induction in microglia in APP/PS1 mice

**Fig. 1:**
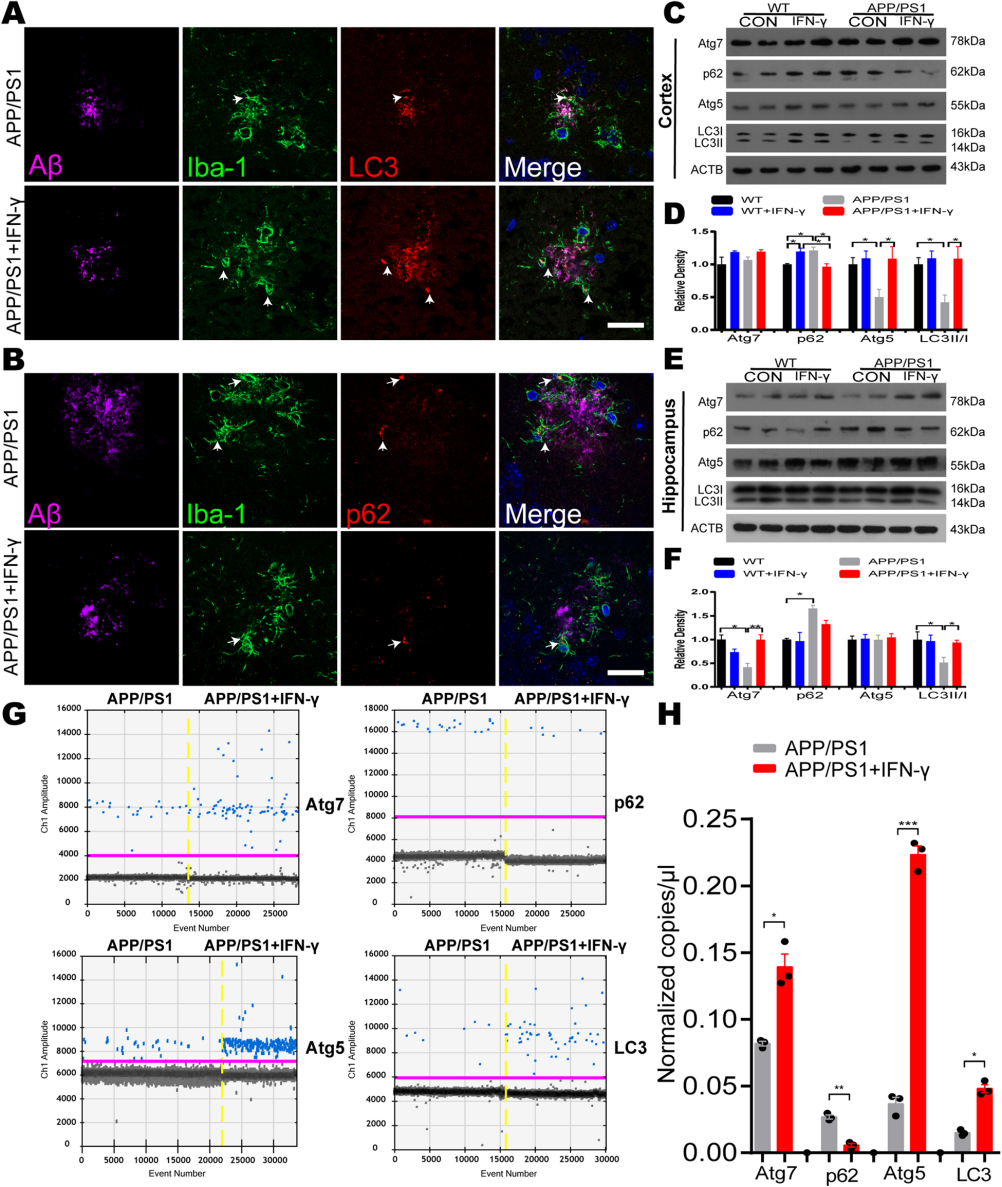
IFN-γ administration induces autophagy in APP/PS1 mice. **a**, **b** Triple immunostaining of mouse brain slices with anti-Aβ (purple), anti-Iba-1 (green) and anti-LC3/anti-p62 (red) antibodies to examine autophagy in APP/PS1 mice treated with IFN-γ or not. Hoechst (blue) was used for nuclear staining. Scale bar = 10 μm. **c**, **d** Western blots of cortical proteins probed with the indicated antibodies. Atg7 expression was not significantly different among the four groups. The p62, Atg5 and LC3II/I levels were different among the four groups. **e**, **f** Western blots of hippocampal tissues probed with the indicated antibodies. Atg5 expression was not significantly different among the four groups. Atg7 and LC3II/I levels were different among the four groups. **g**, **h** Droplet digital-PCR analysis of autophagic genes in the APP/PS1 mice treated with IFN-γ or not. **g** Representative ddPCR reads of cDNA samples from the microglia labeled with TMEM119 antibodies in APP/PS1 mice. **h** ddPCR reads of autophagic genes (Atg7, p62, Atg5 and LC3) copies from the microglia in APP/PS1 mice were represented as bar graph. *P < 0.05, **P < 0.01, one*-*way ANOVA and Bonferroni post hoc test. The results are all shown as the mean ± SEM. (*n* = 3/group).

The previous research showed that IFN-γ elicits autophagy in macrophages^20^, to investigate whether IFN-γ increases autophagy induction in vivo, APP/PS1 mice (8 months old) were intraperitoneally (i.p.) injected with murine IFN-γ (5 × 10^4^ U) and IFN-γ reached the brain within 30 min after i.p. injection (Fig. S1). And then IFN-γ was injected intraperitoneally for 9 days at a dose of 5 × 10^4^ U/day into APP/PS1 mice and WT mice^21^. The control of WT and APP/PS1 received the same volume sterile water. LC3 is a reliable marker of autophagic vacuoles, which are necessary for the elongation of autophagosome membranes and regarded as an indicator of the autophagy induction^22^. The polyubiquitin-binding protein p62 possesses a short LC3 interaction region that helps it directly bind with LC3 and facilitate its degradation. Therefore, any defects in autophagy may lead to the accumulation of p62 in neurodegenerative diseases, such as Alzheimer’s disease^23–25^. In line with a previous report, we found a significant increase in the expression of LC3 and a significant decrease in p62 levels around Aβ plaque in APP/PS1 mice after IFN-γ treatment (Fig. 1a, b). We also examined LC3 and p62 immunoreactivity in plaque-free areas (Fig. S2), the coincident results showed that IFN-γ induced increase in autophagy is Aβ plaque-independent. To determine the role of IFN-γ in autophagy in APP/PS1 mice, we measured the expression levels of Atg7, p62, Atg5 and LC3, which are routinely used to assess overall autophagy induction. As shown in Fig. 1c–f, LC3II/I ratio was significantly decreased in APP/PS1 mice compared with WT mice both in the cortex and the hippocampus; whereas these decreases were restored by IFN-γ treatment. However, p62 levels were higher in the APP/PS1 mice in two brain areas. Interestingly, IFN-γ treatment only restored p62 levels in the cortex but not in the hippocampus. In addition, IFN-γ normalized Atg7 and LC3II/I expression but not Atg5 expression in the hippocampus of APP/PS1 mice. In order to investigate the effect of IFN-γ treatment on microglia, we isolated microglia labeled by TMEM119 antibodies (a special marker for microglia)^26^ by flow cytometry from brains of APP/PS1 mice. Then we determined the expression of the autophagic relevant genes, including Atg7, p62, Atg5 and LC3 by droplet digital PCR (ddPCR). The results showed that IFN-γ treatment increased the expression of autophagic gene in the microglia of APP/PS1 mice (Fig. 1g, h). Besides, we found that IFN-γ significantly increased the expression of the LAMP1 (lysosome associated membrane protein1), which corroborated the enhance of autophagic flux (Fig. S3). These data indicated that IFN-γ treatment enhanced microglial autophagy by degrees in APP/PS1 mice, suggesting a possible mechanism for the autophagy-mediated clearance of Aβ.

### IFN-γ treatment protected BV2 cells from Aβ toxicity by activating autophagy

**Fig. 2:**
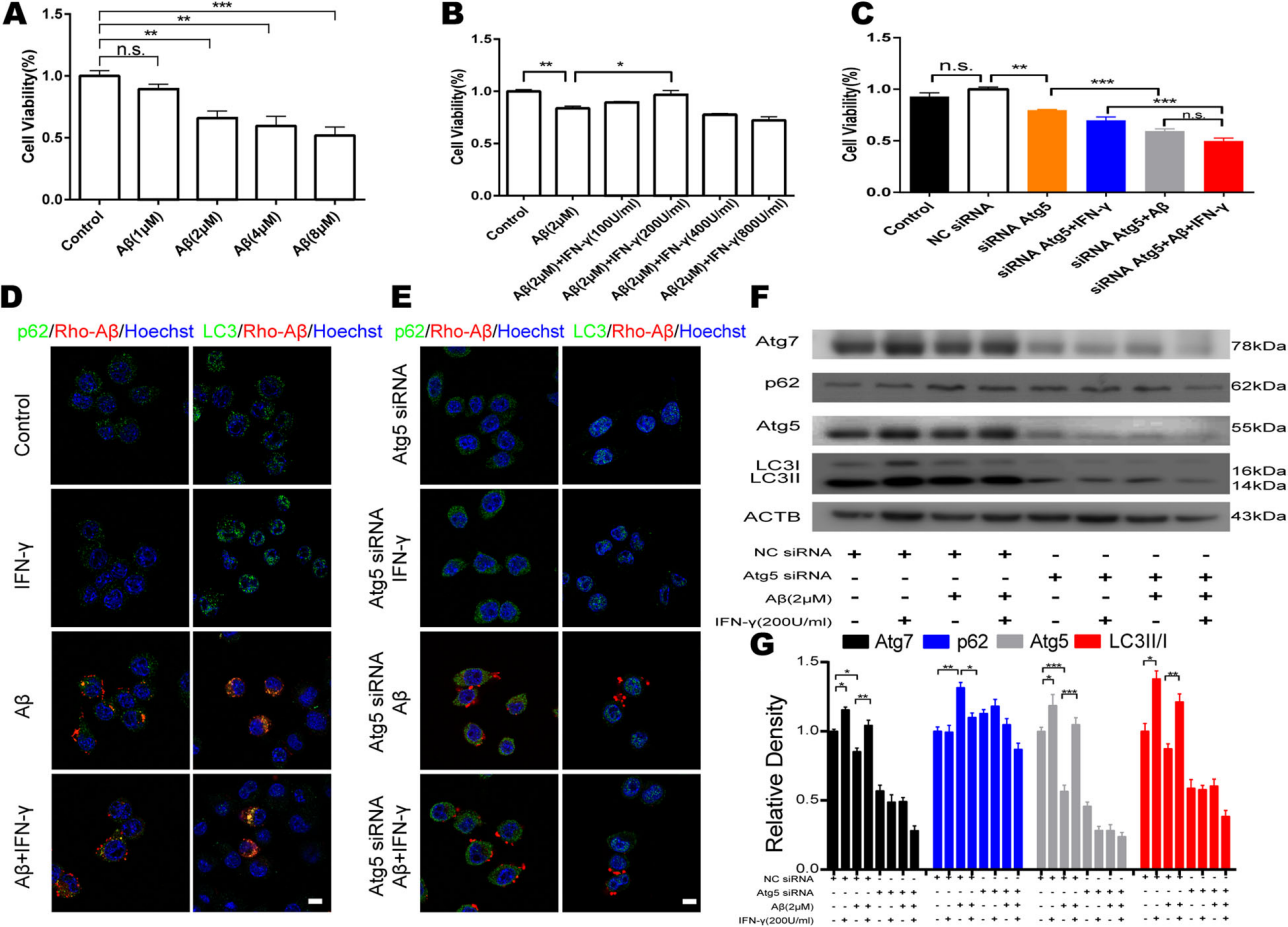
IFN-γ protected BV2 cells from Aβ toxicity by increasing autophagy. **a** BV2 cell viability was measured by a CCK-8 assay after treatment with the indicated concentrations of Aβ for 24 h. **b** The protective effect of IFN-γ on BV2 cells was determined through combined treatment with Aβ (2 μM) for 24 h and various concentrations of IFN-γ for 2 h. **c** IFN-γ could not protect BV2 cells from Aβ cytotoxicity after interference with Atg5. Each treatment was performed in a six-well plate. NC siRNA, negative control siRNA. **d** Immunostaining of BV2 cells with Rhodamine-Aβ (red) and anti-p62/anti-LC3 (green) antibodies to detect autophagy induction in BV2 cells treated with Aβ and IFN-γ. Hoechst (blue) was used for nuclear staining. Scale bar = 10 μm. **e** Immunostaining of Atg5-siRNA-BV2 cells with Rhodamine-Aβ (red) and anti-p62/anti-LC3 (green) to detect autophagy induction in BV2 cells treatment with Aβ or IFN-γ or combined with them. Hoechst (blue) was used for nuclear staining. Scale bar = 10 μm. **f**, **g** Western blots of proteins in BV2 cells probed with Atg7, p62, Atg5 and LC3 antibodies. Changes of autophagy associated proteins in response to Aβ and/or IFN-γ after knockdown of the Atg5 gene. *P < 0.05, ** *P* < 0.01, ***P < 0.001, one-way ANOVA and Bonferroni post hoc test. The results are all shown as the mean ± SEM. Data are representative of three independent experiments with similar results.

To further explore the role of IFN-γ in autophagy induction, we investigated the function of IFN-γ in microglial viability after Aβ treatment in vitro. To verify the cytotoxicity of Aβ in microglia, a CCK-8 assay was performed to assess the viability of differentiated murine BV2 cell, which is a recognized microglia cell line, under Aβ treatment. The results showed that Aβ treatment for 24 h suppressed BV2 cell viability in a dose-dependent manner (Fig. 2a and Fig. S4A). To ascertain the protective effect of IFN-γ against Aβ-induced cell death, BV2 cells were treated with 2 μM Aβ combined with different concentrations of IFN-γ followed by cell viability detection (Fig. S4B). As shown in Fig. 2b, IFN-γ protected BV2 cells from Aβ-induced cytotoxicity at a concentration of 200 U/ml after 2 h of treatment. To characterize the protective effects of the enhancement of autophagy induced by IFN-γ, we designed siRNA to interfere with the Atg5 gene, which plays a critical role in alleviating Aβ deposition and pathology^27^. We designed three siRNAs for the Atg5 gene and verified that Atg5-siRNA-3 had the best inhibitory effect at a concentration of 50 nM (Fig. S5A and B). As predicted, BV2 cell viability was clearly decreased after autophagy blockade induced by interference with Atg5 (Fig. 2c). Next, we examined aspects of cellular morphology and the levels of autophagic proteins. The cellular immunofluorescence and western blot results verified our findings in vivo. Atg5 and Atg7 levels were significantly decreased in BV2 cells treated with Aβ; whereas these effects were reversed after IFN-γ treatment (Fig. 2d–g). However, IFN-γ could not restore Aβ-induced autophagy impairment after interference with the Atg5 gene (Fig. 2d–g). Together with our in vivo data, these results suggested that IFN-γ could protect BV2 cells from Aβ toxicity by enhancing autophagy induction.

### IFN-γ enhanced autophagy associated with the AKT/mTOR pathway in APP/PS1 and BV2 cells

**Fig. 3:**
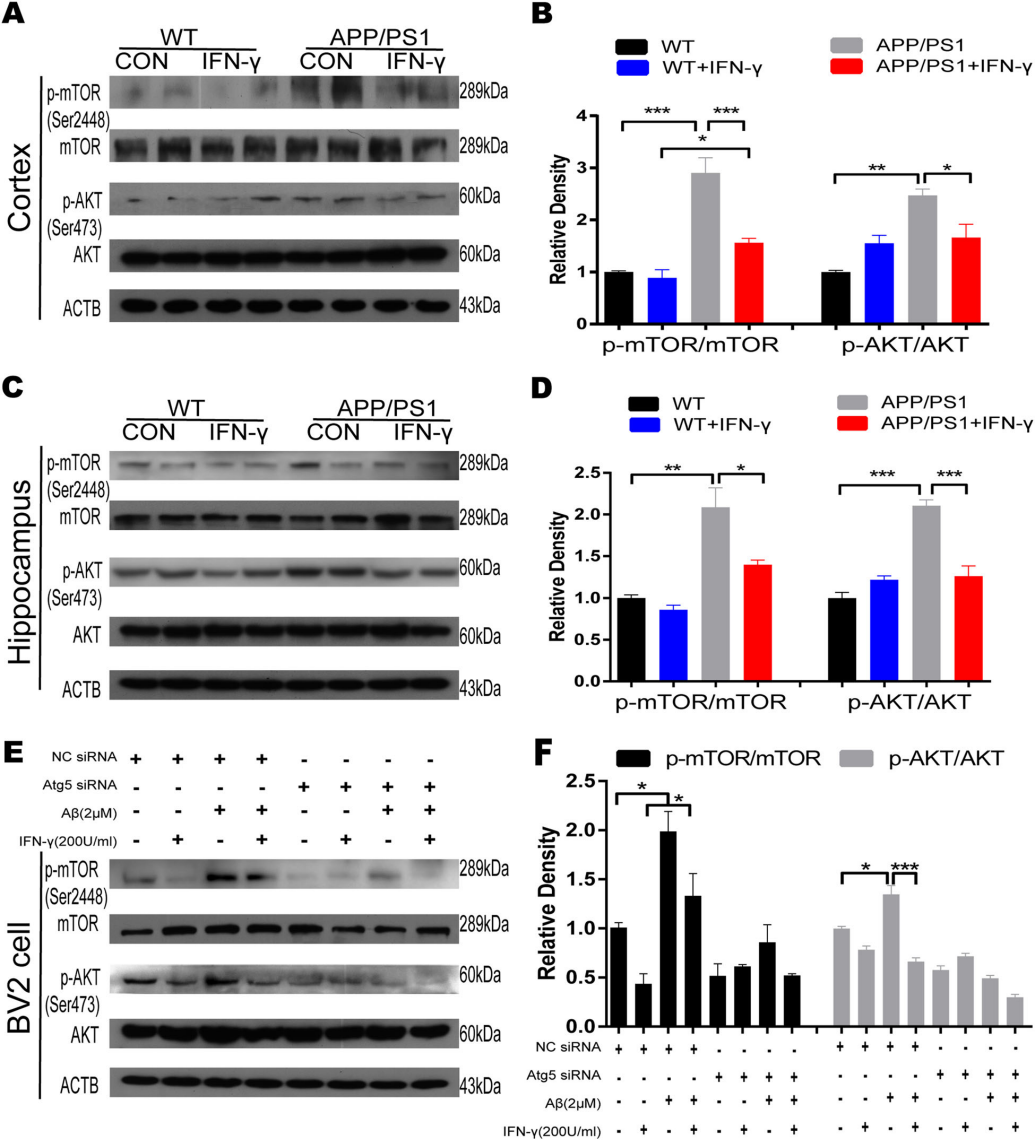
IFN-γ-mediated autophagy is associated with the AKT/mTOR pathway. **a**, **b** Representative Western blots showing the levels of total and phosphorylated AKT/mTOR protein in the cortex of WT and APP/PS1 mice. **c**, **d** Representative Western blots showing the levels of total and phosphorylated AKT/mTOR protein in the hippocampus of WT and APP/PS1 mice. **e**, **f** Representative Western blots showing the levels of total and phosphorylated AKT/mTOR protein in BV2 cells. *P < 0.05, **P < 0.01, ***P < 0.001, one-way ANOVA and Bonferroni post hoc test. The results are all shown as the mean ± SEM. (*n* = 3/group).

Then, we investigated the underlying mechanisms mediating IFN-γ-induced autophagy in vitro. Increasing evidence has confirmed that the AKT/mTOR pathway plays a vital role in neurodegenerative diseases, especially in synaptic development and function^28,29^. The abnormal activation of the AKT/mTOR pathway has commonly been reported in the brains of AD patients, and both its hypoactivation and hyperactivation have been linked to autophagy disruption related to AD pathology^30^. Notably, IFN-γ is an important mediator of inflammation and may activate the AKT/mTOR signaling pathway^31^. To test whether IFN-γ treatment protected BV2 cells from Aβ toxicity by enhancing autophagy through suppressing the AKT/mTOR pathway, we measured the levels of p-mTOR, mTOR, p-AKT, AKT in the microglial BV2 cell line and APP/PS1 mice by Western blot. Our results showed that p-mTOR and p-AKT were clearly inhibited by IFN-γ in the cortex and hippocampus of APP/PS1 mice and that their levels were similar to those observed in the controls (Fig. 3a–d). In vitro, IFN-γ also prevented the increase in p-AKT and p-mTOR caused by Aβ (Fig. 3e, f). Overall, IFN-γ enhanced microglial autophagy, which was, at least in part, linked to AKT/mTOR signal suppression.

### IFN-γ treatment altered the cerebral amyloid-β load in APP/PS1 mice

**Fig. 4:**
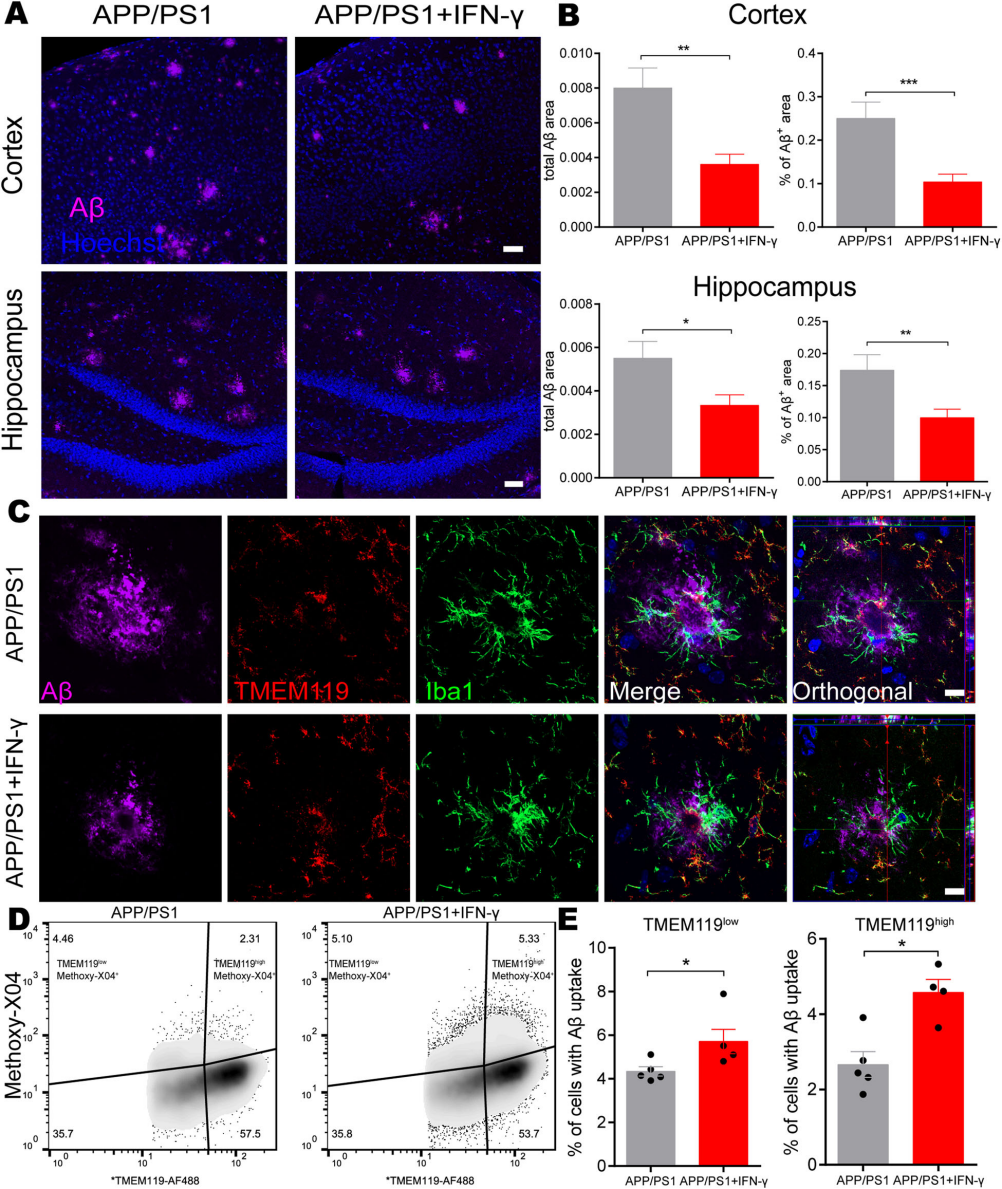
IFN-γ administration promotes microglial clearance of Aβ. **a** Representative image of staining with anti-Aβ (purple) and Hoechst (blue) in the cortical and hippocampal areas of APP/PS1 mice treated with IFN-γ or not. Scale bar = 50 μm. **b** Quantification of the Aβ burden in the cortex and hippocampus in APP/PS1 mice. *P < 0.05, **P < 0.01, ***P < 0.001. Data were analyzed by Student’s *t*-test and are presented as the mean ± SEM. (*n* = 6/group) **c** Representative images of staining with anti-Aβ (purple), TMEM119 (red), Iba-1 (green) and Hoechst (blue) in APP/PS1 mice treated with IFN-γ or not. Scale bar = 10 μm. **d**, **e** IFN-γ enhances the phagocytic activity of microglia. Representative density plots and quantification show the percentages of microglia (TMEM119) containing Aβ (Methoxy-X04) in APP/PS1 mouse brains after IFN-γ treatment. APP/PS1: *n* = 5 mice; APP/PS1 + IFN-γ: *n* = 4 mice. *P < 0.05. Data were analyzed by Student’s *t*-test and are presented as the mean ± SEM.

As we had observed that peripheral IFN**-γ** administration obviously facilitated autophagic induction in microglia BV2 cells, and microglia are considered the principal immune effector phagocytic cells involved in autophagy in the brain^32^, we continued to examine whether this peripheral immune activation was able to affect Aβ phagocytosis by resident microglia in APP/PS1 mice. To determine the role of IFN-γ in Aβ protein clearance in the brain, we stained brain sections from APP/PS1 mice with Aβ-specific antibodies. We observed that the Aβ plaque burden in IFN-γ-administered APP/PS1 mice was significantly decreased both in the cortex and hippocampus compared with those in control mice (Fig. 4a, b). In addition, in the present study, we observed that CX3CR1 positive cells (mainly microglia) tended to encircle and phagocytose Rhodamine-Aβ (white arrows) in the somatosensory cortex by using the two-photon imaging system (Supplementary Video S1), and in Supplementary Video S2, the two-photon imaging results showed that CX3CR1 positive cells merged with Aβ plaque-labeled with Methoxy-X04, a fluorescent dye could cross the BBB and specifically label Aβ^33^, and tended to phagocytose Aβ in APP/PS1;CX3CR1-GFP^+/−^ mice. The immunofluorescence results also confirmed it (Fig. S6). Our data show there are much more Iba-1 positive macrophages and much less TMEM119 positive microglia in Aβ areas (Fig. 4c), supporting decreased TMEM119 expression around Aβ burden. What’s more, in Fig. 4c, the Iba-1^+^/TMEM119^−^ cell might be the monocyte-derived macrophages, which suggests that the result in the brain caused by IFN-γ could be mediated by the infiltrating monocytes and not only microglia.

To directly demonstrate and quantify that IFN-γ-mediated Aβ phagocytosis by microglia, the APP/PS1 mice were intraperitoneally injected with Methoxy-X04. Flow cytometry analysis detected Methoxy-X04-labeled Aβ and TMEM119 AF-488-labeled microglia from the brains of APP/PS1 mice. The data revealed that IFN-γ injection further increased both the proportion of TMEM119^high^ cells and TMEM119^low^ cells exhibiting Methoxy-X04 fluorescence (Fig. 4d, e). The increase of TMEM119^high^ cells might be caused by IFN-γ treatment-induced peripheral immunity. These findings suggest that IFN-γ treatment significantly increases Aβ phagocytic uptake by microglia. To investigate whether IFN-γ has the effect on the APP processing, we measured the expression of APP (Aβ precursor protein) and BACE (β-site APP-cleaving enzyme) in the cortex and the hippocampus of APP/PS1 mice. We found there were no significant changes in APP and BACE levels in APP/PS mice either treated with IFN-γ or not (Fig. S7). Taken together, our results demonstrate that clearance of the cerebral Aβ burden is correlated with microglial autophagy facilitation in IFN-γ-treated APP/PS1 mice, suggesting that IFN-γ administration increases phagocytic Aβ uptake by microglia, rather than affects APP processing.

### IFN-γ treatment alleviated cognitive deficits and synaptic impairments in APP/PS1 mice

**Fig. 5:**
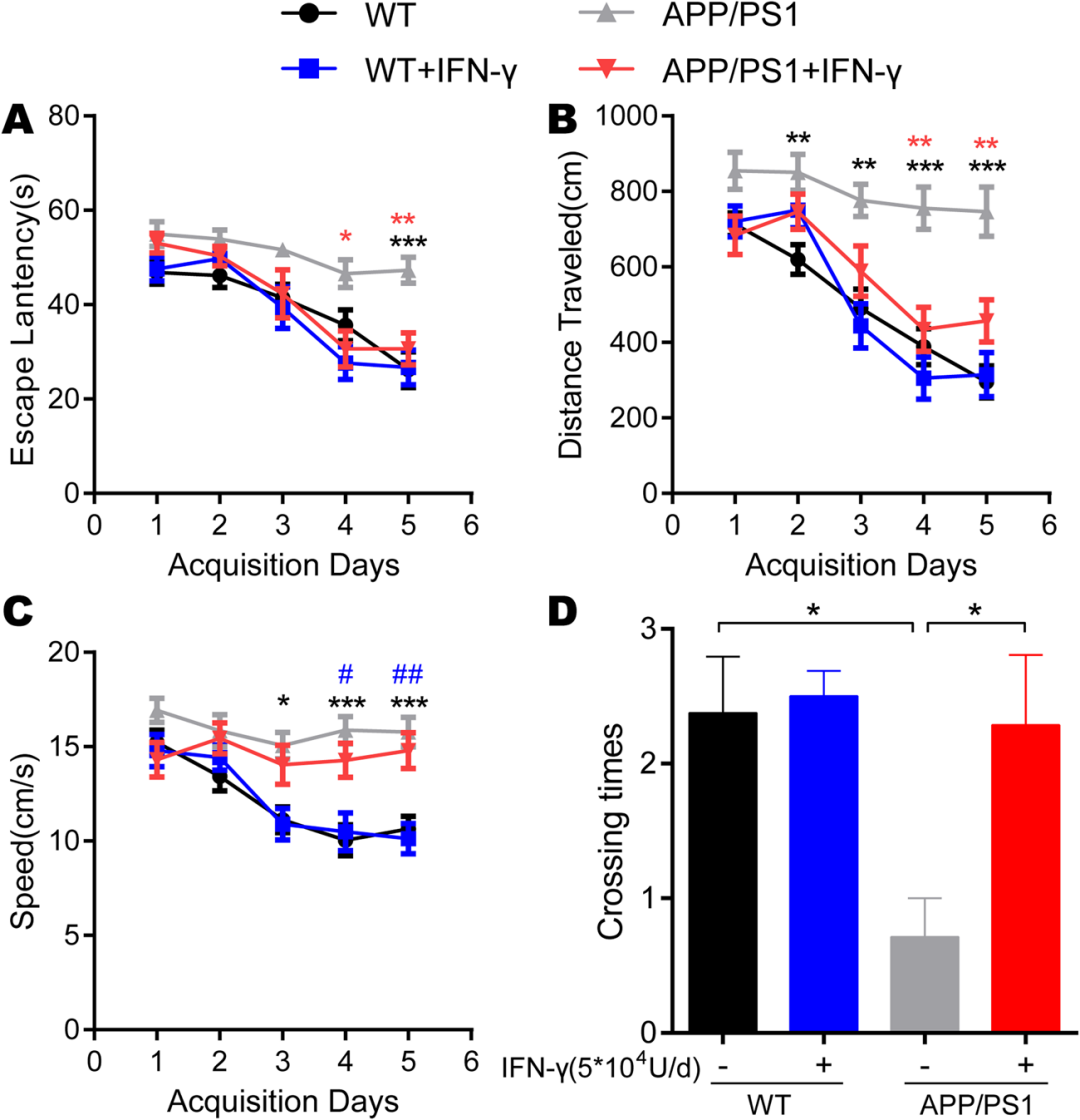
IFN-γ administration improved cognitive deficits in APP/PS1 mice. **a**, **b** Learning curves of mice trained in the spatial reference version of the Morris water maze (*n* = 9/group). The escape latency and the distance traveled to find the hidden platform were plotted against the days of training. Each day represents the average of four consecutive training trials. Both measurements indicated that all groups significantly improved over the 5 days. *P < 0.05, **P < 0.01, ***P < 0.001 vs APP/PS1 group (two-way ANOVA with repeated measure and Bonferroni post hoc test). Specifically, the APP/PS1 group was significantly impaired compared to the other three groups at days 4 and 5 in terms of escape latency and at days 2–5 in terms of the distance traveled. **c** There was no difference in the speed between the APP/PS1 + IFN-γ group and the control group. *P < 0.05, ***P < 0.001 vs APP/PS1 and ^#^*P* < 0.05, ^##^*P* < 0.01 vs APP/PS1 + IFN-γ (two-way ANOVA with repeated measure and Bonferroni post hoc test). **d** In the probe trial phase, the number of passes across the former location of the escape platform during the MWM test are shown. *P < 0.05 vs APP/PS1 (one-way ANOVA and Bonferroni post hoc test). The results are all shown as the mean ± SEM.

**Fig. 6:**
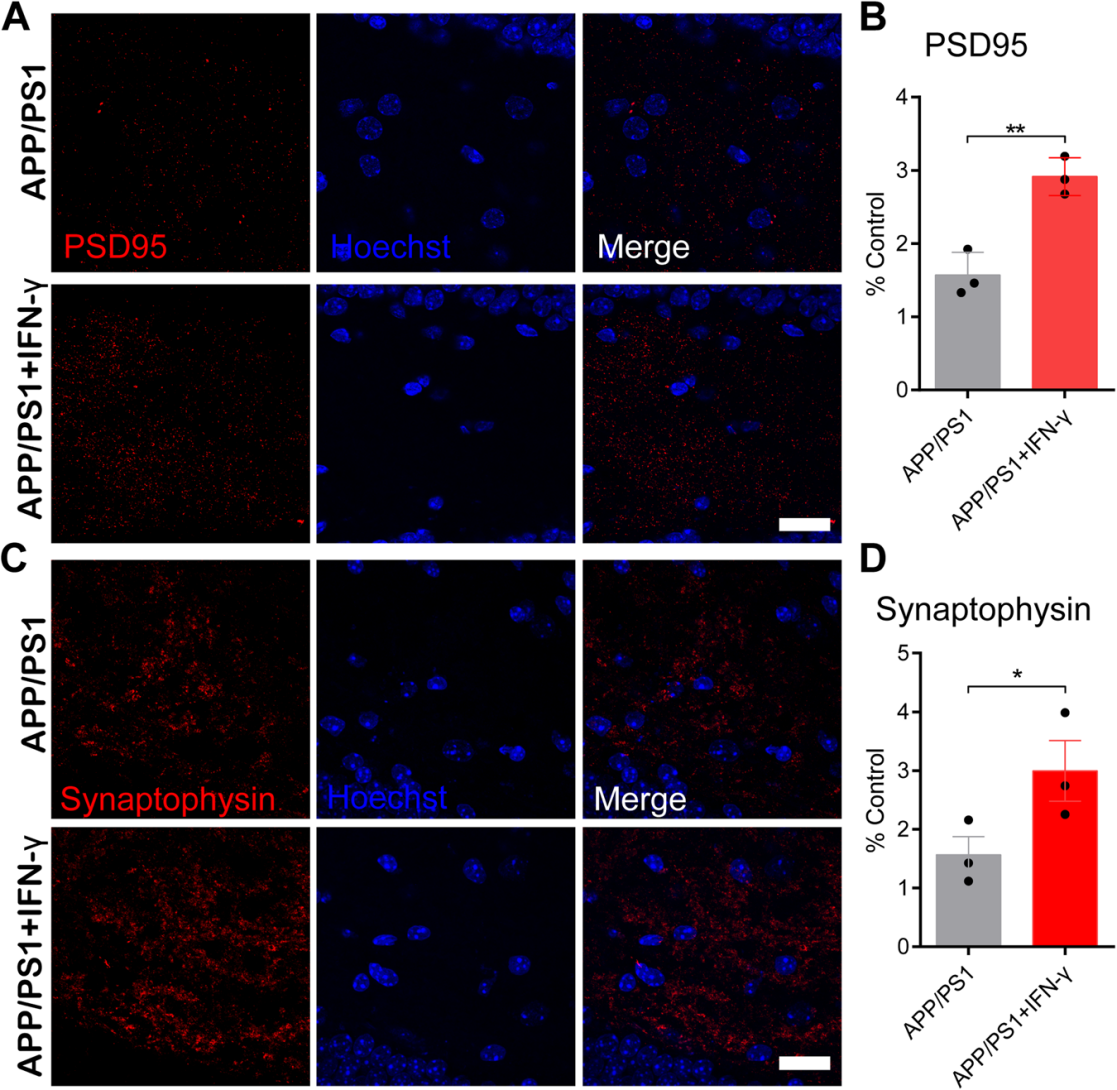
IFN-γ interacted with neurons and increased the expression of PSD95 and synaptophysin. **a**–**d** IFN-γ enhances the expression of PSD95 and synaptophysin in the hippocampus. **a**, **c** Representative images of staining with anti-PSD95/synaptophysin (red) antibodies and Hoechst stain (blue) in APP/PS1 mice and APP/PS1 + IFN-γ mice. Scale bar = 20 μm. **b**, **d** Quantification of PSD95 and synaptophysin in treated mice. *P < 0.05, **P < 0.01. Data were analyzed by Student’s *t*-test and are presented as the mean ± SEM. *n* = 3/group.

Next, we investigated the effect of IFN-γ on hippocampus-dependent spatial learning and memory abilities by using the Morris water maze test and found a significant improvement in spatial cognitive function in APP/PS1 mice after 9 days of IFN-γ administration relative to the genotype-matched controls, and we found that the APP/PS1 mice achieved performance similar to that of WT mice. The improvement was reflected in the escape latency, traveled distance and crossing times (Fig. 5a–d). Although there is no significant difference in time spent in the target quadrant, the result showed the tendency that IFN-γ treatment reversed the decrease of the time that APP/PS1 mice spent in the target quadrant (Fig. S8). Notably, there were no obvious differences in these parameters between the IFN-γ-treated WT mice and WT controls, suggesting that a genotype-dependent effect or ceiling effect plays a role in IFN-γ treatment-induced cognitive improvement. During the Morris water maze, no differences in swimming speed were observed between groups with the same genotype (Fig. 5c). Immunofluorescence analysis of synaptophysin and PSD95 showed a significant increase in the cortex and hippocampal regions (Fig. 6a–d), which could be another reason that IFN-γ improved cognitive defects in APP/PS1 mice. We concluded that the infiltration of IFN-γ may encourage crosstalk between neurons expressing synaptic proteins and resident microglia to mediate Aβ phagocytosis.

In addition, no abnormal, disease-like behavior was observed in the APP/PS1 transgenic mice during IFN-γ injection, as reflected by the data from the OFT (Fig. S9A, B). The body weights of the mice, which were monitored every day, remained steady, and no significant differences were observed between the IFN-γ-injected group and the controls (Fig. S9C).

## Discussion

In this study, we showed that the intraperitoneal injection of IFN-γ enhanced microglial autophagy, promoted Aβ plaque clearance both in the hippocampus and the cortex, and improved cognitive ability in 8-month-old APP/PS1 mice. Furthermore, we confirmed that Atg5-dependent autophagy induction played a vital role in the IFN-γ-induced protection of BV2 cells from Aβ cytotoxicity, which involved the suppression of AKT/mTOR signaling. Based on the protective effects of IFN-γ on synaptic functioning observed in the APP/PS1 mouse model, our findings suggested that peripheral IFN-γ administration might provide a potentially effective immunotherapy strategy for AD.

IFN-γ is a pleiotropic cytokine that is secreted by Th1 cells, dendritic cells, and NK cells and participates in innate and/or adaptive immune responses against viral infections, bacterial infections and antigen presentation^34^. However, IFN-γ treatment is a double-edged sword, the previous research showed that the anti- and pro-tumorigenic activities of IFN-γ treatment are dependent on the cellular and molecular environment^35^. Besides, prolonged treatment with IFN-γ would induce erythema nodosum leprosum in lepromatous leprosy patients^36^. So there are the limitations of prolonged treatment with IFN-γ in terms of side work. IFN-γ-targeted research has gradually involved the examination of neurodegenerative disorders, such as Alzheimer’s disease. Even though the conclusions were inconsistent, they revealed a clear role of IFN-γ with respect to Aβ burden phenotypes and cognitive performance in the AD mouse model^18,37,38^. Similarly, accumulating evidence over the past decade supports the idea that pro-inflammatory cytokines play a vital role in the pathogenesis of Alzheimer’s disease. Conversely, blocking anti-inflammatory cytokine signaling in preclinical AD mouse models provides a novel concept for treatment^39^. Here, our results showed that peripheral IFN-γ administration promoted the Aβ-clearing capacity of microglia, which were activated and recruited to Aβ plaques. Consistently, activation of the innate immune system and stimulation of TLR4 by LPS have been shown to clearly reduce AD-like pathology, including Aβ deposition and Tau phosphorylation^40,41^. Moreover, we showed that autophagy impairment was restored in the microglia of APP/PS1 mice by IFN-γ treatment. Mechanistically, IFN-γ-induced Aβ phagocytosis and the protection of BV2 cell viability were both prevented by using Atg5-siRNA in our in vitro experiments. Therefore, together with the results of other studies^5,18^, we showed that autophagy restoration by IFN-γ is required for the degradation of Aβ protein in APP/PS1 mice.

Autophagy is a multistage process that involves a cascade of events linked to a series of signaling pathways^42^. Therefore, we further measured several autophagic signaling molecules and related proteins. Atg5, Atg7 and LC3II/I expressions were significantly restored to normal levels, which were impaired in Aβ-treated BV2 cells. Knocking down the Atg5 gene by siRNA partly prevented Aβ clearance in BV2 cells. In vivo, Atg5 levels in the cortex but not in the hippocampus were obviously decreased, and this is associated with autophagy impairment in APP/PS1 mice. Actually, contrary to our assumptions, alterations of autophagy protein Atg5 and Atg7 are inconsistent both in the cortex and hippocampus of APP/PS1 mouse model. Although a great many studies reported autophagy proteins, in particularly, Atg5 and Atg7, are disorganized in AD patients and/or mouse models, increasing or decreasing in Atg5 and Atg7 is still inconclusive in AD mouse models. For example, Beclin-1, also known as Atg6, is a representative autophagy protein with conflicting reports in APP transgenic mouse models of AD^43–45^. Therefore, it is understandable that inconsistent results were observed in different brain areas. In vitro test, IFN-γ fails to modulate LC3II/I, p62 and Atg7, when upstream autophagy protein Atg5 was silenced. Although there is an increase of p62 in the WT mice after IFN-γ treatment, Atg5-Atg12 conjugation system, including Atg5, Atg7 and the ratio of LC3II/I remain constant in the cortex of WT mice treated with IFN-γ or not. Therefore, autophagy flux may not have been initiated. However, it is not ruled out that some autophagy-related changes with age happens at the age of 8 months, because on related data interpret decreased autophagy activity during aging in the present study^46^.

A recent report showed that a 5 × FAD mouse model that contains a conditional deletion of Atg5 in microglia showed Aβ deposition and pathology both in the cortex and hippocampus compared with the control littermates^27^. Besides, it reports that autophagy protein LC3 can be recruited to endosomes containing Aβ in BV2 microglia in vitro, which is dependent on Atg5. Therefore, it is understandable that there is co-localization of LC3 and Aβ in the APP/PS1 mouse model. And it might be the reason that there is the difference in the staining pattern between plaque-free and plaque-associated areas. In our research, we confirmed that Atg5 and Atg7 are maintained at a constant level in the hippocampus and cortex in APP/PS1 mice, whether or not they are treated with IFN-γ. These findings suggested that there were differences between the experiments in vivo and in vitro. In line with the results in a previous report, Atg5/Atg7-independent alternative pathways, such as that involving Rubicon, which is a key component of a noncanonical pathway of autophagy that regulates Aβ deposition, are also involved in mammalian macroautophagy^27,47–49^. Thus, Atg5/Atg7-independent pathways, especially in different AD mouse models, remain to be determined in the future.

What are the downstream mechanisms that mediate IFN-γ signaling to induce microglial autophagy? PI3K/AKT/mTOR signaling is required for autophagy induction by IFN-γ stimulation in macrophage cell lines^20^. Therefore, we focused on the PI3K/AKT/mTOR pathway, which is the major regulator of autophagy in different cell types. IFN-γ treatment normalized the levels of p-AKT and p-mTOR, which were abnormally activated both in the vitro and vivo experiments, indicating that AKT and mTOR may be the downstream kinases involved in IFN-γ signal transduction. In line with this result, a recent study reported that alborixin clears Aβ by inducing autophagy through the PTEN-mediated inhibition of the AKT pathway^50^.

Importantly, p-AKT and p-mTOR can inhibit autophagy by inhibiting Atg5 expression. Unexpectedly, the knockdown of Atg5 by siRNA clearly decreased p-AKT and p-mTOR in both Aβ-treated and Aβ + IFN-γ-treated BV2 cells, suggesting that there was positive feedback between Atg5 and the phosphorylation of AKT/mTOR. Under normal physiological conditions, a certain level of Atg5 maintains proper autophagy, which is essential to many pivotal host functions, including the clearance of protein fragments and the resolution of inflammation^51^. In neurodegenerative diseases, such as AD, p-AKT and p-mTOR are activated by Aβ and impair autophagy induction. After IFN-γ treatment, decreases in p-AKT and p-mTOR reduced the inhibition of Atg5, restored autophagy impairment, and ultimately promoted Aβ clearance. The high expression of Atg5 exerted a positive effect on p-AKT and p-mTOR, which in turn inhibited Atg5. Therefore, long-term therapy might be needed for the effects of IFN-γ in AD due to this positive feedback mechanism.

Synaptophysin is the major integral membrane protein in synaptic vesicles. Postsynaptic density 95 (PSD95), which is the major scaffolding protein that contributes to excitatory PSD and is a potent regulator of synaptic strength, promotes synapse stability^52^. In the present study, we observed that IFN-γ enhanced the expression of PSD95 and synaptophysin in the hippocampus of APP/PS1 mice, which is in line with previous findings showed that neurogenesis was enhanced by IFN-γ in a mouse model of AD^53,54^. Notably, autophagy induced by IFN-γ is also necessary for memory formation and is shown to restore age-related memory decline in one recent study^55^. Thus, IFN-γ treatment might be a valid approach to restore synaptic plasticity and neuronal function in an AD mouse model.

**Fig. 7:**
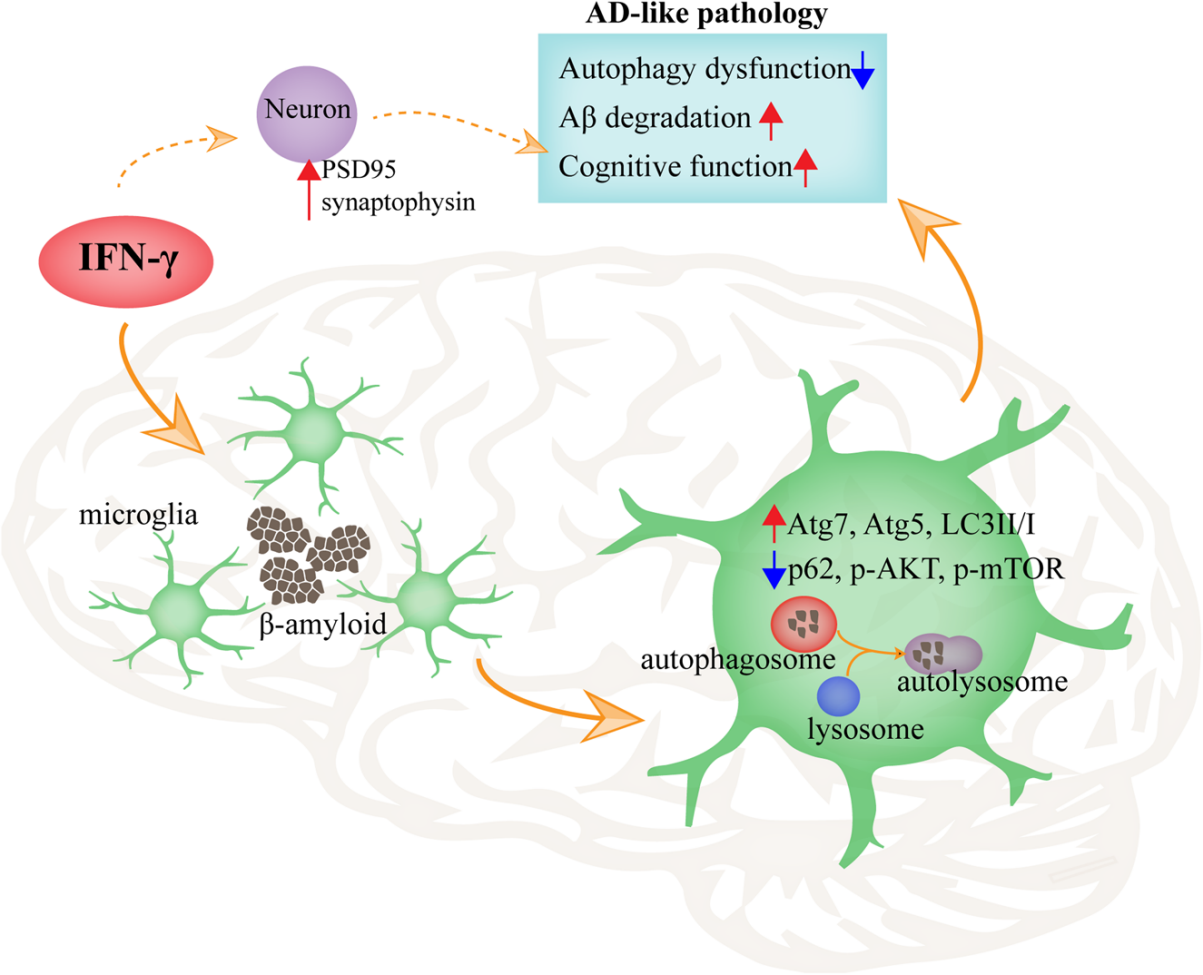
Graphical abstract of this study. The proposed role of IFN-γ-mediated autophagy on AD-like pathology in a murine model. IFN-γ promotes microglial activation, upregulates autophagy in microglia, promotes Aβ clearance, reduces the dysfunction of autophagy, and improves memory deficits.

Our data indeed confirmed that the decrease in Aβ burden and the improvement of cognition in APP/PS1 mice are associated with an increase in autophagy following IFN-γ treatment. Additionally, Aβ-targeted immunotherapy has been proven to be an efficacious strategy for clearing Aβ deposits from the brain, but it was shown to induce brain inflammation in preclinical and clinical trials^56^. These observations warrant further testing of IFN-γ to explore the possibility of maintaining the beneficial effects without the side adverse effects. In addition, IFN-γ has been used in the clinic and has been shown to be a safe and economic treatment^57–59^. Our results suggested that, regardless of increases in autophagy or synaptic transmission, IFN-γ is beneficial for cognition and memory. Based on these lines of evidence, we have suggested that the activation of IFN-γ-mediated autophagy would be a promising therapeutic approach for AD (Fig. 7).

In summary, the present study demonstrated that IFN-γ treatment could be a safe, valid and feasible therapeutic approach for AD, as it facilitates Aβ clearance by restoring autophagy. Further studies will be conducted to explore whether IFN-γ could increase neuronal autophagy to enhance synaptic transmission and to improve memory function.

## Materials and methods

### Animals

Female, 8-month-old APP/PS1 mice co-expressing APP (human mutant (KM670/671 L) amyloid precursor protein) and PS1 (mutant (M146L) presenilin 1), age-matched wild-type (WT) mice were purchased from Guangdong Medical Laboratory Animal Center (GMLAC, Guangzhou, China). CX3CR1-GFP^+/+^ transgenic mice (B6.129P-CX3CR1tm1Litt/J) were purchased from the Jackson Laboratory (stock no. 005582). All animals were generated with the C57BL/6 background and raised in specific pathogen-free conditions with 12-h light/12-h dark cycle at 22 ± 1°C and with free access to food and water. According to the random number table, 36 mice were randomly divided into four groups: WT, WT + IFN-γ, APP/PS1 and APP/PS1 + IFN-γ. The care and handling procedures of animals were approved by the Institutional Animal Ethics Committee of Sun Yat-sen University.

### BV2 cell line

The BV2 murine microglial cell line was cultured in high glucose DMEM (Dulbecco’s modified Eagle medium) (Gibco-BRL, 11965-092) with 10% FBS (fetal bovine serum) (Gibco-BRL, 10099-141) and 1% penicillin-streptomycin (Life Technologies, Inc., 15140122) in an incubator (Thermo Fisher Scientific, MA, USA) with 5% CO_2_ and 95% humidity at 37°C.

### Preparation of Aβ fibrils

Rhodamine-amyloid β1-42 (Rho-Aβ) was purchased from Nanjing Peptide Biotech Ltd. (NJP40319), dissolved in DMSO to a final concentration of 500 µM and then stored at −80 °C until use. To maintain the condition of fibrils, medium (DMEM with 10% FBS) was added and the final concentration was 50 µM. The solution was then incubated for 24 h at 37 °C before using.

### RNA interference and transfection

Three siRNAs targeting Atg5 and one negative control siRNA (NC siRNA) were purchased from RiboBio (Guangzhou, China). BV2 cells (5 × 10^5^ per well) were seeded into six-well plates and grew to 50% confluence. Before transfection, the original culture medium was removed, the cells were washed with DMEM medium. In all, 5 μl Atg5 siRNA or NC siRNA was dissolved in 120 μl DMEM medium, and 5 μl Lipofectamine TM 3000 (Invitrogen, L3000008) was dissolved in 120 μl DMEM medium; then mixed them up to the final volume of 250 μl. The Lipofectamine-siRNA mixture was incubated at room temperature for 15 min, then the mixture was added to each well with additional 1750 μl DMEM medium. After 6 h, the medium was removed and added 2 ml of complete medium to each well. We optimized the time of cell harvest and the siRNA concentration for transfection by three different concentrations of siRNA (10 nM, 25 nM, and 50 nM) and an optimized 24 h time frame. The efficiency of the siRNA down-regulating the expression of Atg5 mRNA was determined by real-time quantitative PCR analysis.

### Morris water maze (MWM)

One week after the last injection, the behavioral testing was performed during the light period in the 24 h light/dark cycle. The apparatus used a circular metal pool (80 cm in diameter) that filled with opaque water at 22 ± 1 °C. There are four quadrants in the maze. In the center of one quadrant, a clear escape platform (8 cm in diameter) was located 1.0 cm below the water surface. In order to ensure the normal swimming ability and eyesight of the experimental mice, the day before the experiment, the platform was exposed to the water to ensure mice could see it. If mice swam directly to the platform after put into the water, the swimming ability and eyesight of mice would be fine. At the first day, mice were trained to find the escape platform under the help of the maze cues. During the task acquisition, mice need to swim four trials per day for five consecutive days. They were placed into the maze in a random fashion. When mice find and climb onto the platform, the escape latency was recorded. If mice failed to find the platform within the allowed time (60 s), they were placed on the platform for 15 s manually. The intertrial interval of each mouse was 5 min. At the 6th day, spatial probe trials were conducted, we removed the platform and mice swim for 60 s to determine their search bias. The data were recorded by the automated tracking system (MT-200, Chengdu, China). All experiments were performed blind to genotype and coincident with the guidelines of the National Health and Medical Research Council (NHMRC).

### Open field test (OFT)

The mice were individually placed in the center of open field arena (50 × 50 × 50 cm), there is an illumination on the top of the equipment. The spontaneous locomotor activity of each mice was recorded during 10 min by the TopScanTM 2.0 system (Clever Sys. Inc.). The time spent in the area and total distance were recorded automatically.

### ELISA

30 min after IFN-γ injection, the plasma was separated from trunk blood at room temperature for 2 h and by centrifugation at 3000 rpm for 15 min, then subsequently stored at −80 °C until use. The cortex and hippocampus were immediately removed from the brain, then processed into tissue homogenate with cold RIPA buffer (Beyotime Biotechnology, Wuhan, China) containing phenylmethylsulfonyl fluoride (PMSF) by using a homogenizer. After centrifugation (4 °C, 12000 rpm, 15 min), the supernatant was subdivided and subsequently stored at −80 °C for further measurements. The IFN-γ levels in plasma and brain tissue were measured using a murine recombinant IFN-γ ELISA kit (Neobioscience, EMC101g, Guangzhou, China) according to the manufacturer’s instructions.

### Stereotaxic brain injection and vein tail injection

Six-month-old CX3CR1-GFP^+/−^ mice were injected with 2 μl of 100 µM Rho-Aβ at a speed of 0.4 µl/min with a pump using a 10 µm diameter needle (Thermo Fisher). Unilateral stereotaxic injections were performed by using the stereotaxic apparatus (Reward Instruments) in the left lateral ventricles (rostro-caudal −0.75 mm; lateral −1.0 mm; depth −2.5 mm). The needle was gently removed 5 min after injection. Methoxy-X04 was injected into the age-matched APP/PS1; Cx3cr1-GFP^+/−^ mice through tail vein (50 μl, 5 mg/kg). All of the optical living imaging was recorded by two-photon microscope 1d after receiving two of the different injections.

### In Vivo Transcranial Two-Photon Imaging

The role of microglia in Aβ degradation was further examined in vivo. We stereotaxically injected 2 μl of 100 µM Rho-Aβ into the lateral ventricles of CX3CR1-GFP^+/−^ mice. GFP-positive microglia and rhodamine (Rho)-labeled plaques were imaged through a thinned skull preparation^60^. The transgenic mice were anesthetized with 20% urethane filtered with a 0.22 µm filter (Millipore), and the skull was exposed with a midline scalp incision. An ~1-mm-diameter region of the skull over the somatosensory cortex was thinned with a high-speed drill and a microsurgical blade to a final thickness of ~40 μm. To reduce respiration-induced movement artifacts, the skull was glued to a stainless steel plate. A mode-locked laser (MaiTai HP-OL/Insight DS-OL, Spectra-Physics) was used for two-photon excitation and set to 920 nm for the imaging of GFP, 1120 nm for the imaging of Rho and 835 nm for the imaging of Methoxy-X04 using an in vivo two-photon imaging system (Olympus FVMPE-RS). Images were obtained by using a 1.05 numerical aperture, 25× water-immersion objectives and 2.0× digital zoom. The stack was typically obtained 150 μm below the pial surface^61^.

### qRT-PCR

To verify the efficiency of siRNA, total RNA was extracted from cells using a HiPure Total RNA Mini Kit (Magen, R4111-02, Guangzhou, China). The remaining total RNA was used to synthesize cDNA with HiScript® II Q RT SuperMix (Vazyme, R223-01, Nanjing, China) for the validation of Atg5 by qRT-PCR. qRT-PCR was carried out with the ChamQTM SYBR® qPCR Master Mix (Vazyme, Q311-02, Nanjing, China), Atg5 primers was using the iQ5 MYiQ Real-Time PCR CFX-96 system (Bio-Rad USA). The expression levels of Atg5 were normalized to those of GAPDH. The Cq (quantification cycle) values were obtained by the Lightcycler instrument, and the delta Cq values were used to determine the relative gene expression compared with those of the control.

### CCK-8 assay

Cell proliferation was measured using a CCK-8 assay kit (Dojindo, Japan). BV2 cells (10^4^ cells/well) were incubated in 96-well plates in the presence of different concentrations of Aβ (1 μΜ, 2 μΜ, 4 μΜ, and 8 μΜ) for 2 h, 6 h, 12 h and 24 h, respectively, and different concentrations of IFN-γ (100 U/ml, 200 U/ml, 400 U/ml, and 800 U/ml) for 2 h, 6 h, 12 h, and 24 h, respectively. Then, 10 µl of CCK-8 solution was added to each well, and the plates were incubated for 1 h at 37 °C. The optical density of each well was measured at 450 nm by using a microplate reader. Three independent experiments were performed.

### Immunofluorescence (IF)

The coronal brain slices were blocked in 1% (w/v) BSA with 0.25% Triton X-100 (Sigma) at 37 °C for 1 h, then incubated with primary antibodies at 4 °C overnight. The next day, the slices were washed three times in PBS for 5 min each and incubated with corresponding secondary antibodies at 37 °C for 2 h. The slices were stained with Hoechst nuclear staining (Enzo Life Sciences, H3570, 1:1000) for 1 min, mounted with Immu-Mount (Thermo). The primary antibodies included: mouse anti-Aβ (Sigma-Aldrich, A5213, 1:2000), goat anti-Iba-1 (Abcam, ab5076, 1:1000), rabbit anti-LC3 (Cell Signaling Technology, 3868, 1:1000), rabbit anti-p62 (Cell Signaling Technology, 5114, 1:1000), rat anti-LAMP1 (Abcam, ab25245, 1:500,), rabbit anti-TMEM119 (Abcam, ab209064, 1:1000), anti-NeuN (Abcam, ab104224, 1:1000), mouse anti-PSD95 (Sigma-Aldrich, P246, 1:1000), and mouse anti-synaptophysin (Sigma-Aldrich, S5768, 1:1000). The following fluorescent secondary antibodies were used: Alexa Fluor 488-conjugated donkey anti-goat, Alexa Fluor 488-conjugated donkey anti-rabbit, Alexa Fluor 555-conjugated donkey anti-rabbit, Alexa Fluor 594-conjugated donkey anti-Rat and Alexa Fluor 647-conjugated donkey anti-mouse (Invitrogen, 1:1000). Images of stained brain slices were obtained using a confocal microscope (LSM800; Carl Zeiss), and each specific signal used the same parameters. For the image analysis, LSM 800 confocal laser scanning microscope was used to capture the images of each section with the same parameters to avoid potential technical artifacts. Measurements were performed on a continuous equidistant of five coronal slices spaced 200 μm apart. ImageJ was used to quantify the staining areas of interested area of each image. For the quantification of Aβ staining, we quantified the percentage of the surface area and total area of Aβ plaques in the cortex and hippocampus of the APP/PS1 mice either treated with IFN-γ or not.

### Western blot analysis

Cell lysates of brain tissues (cortex and hippocampus) and cultured BV2 cells were prepared using protein lysis buffer (Beyotime Institute of Biotechnology, P0013C). The protein concentration was determined using a BCA protein assay kit (Beyotime Institute of Biotechnology, P0012). A total of 10 μg of protein was separated by 10% SDS-PAGE and transferred to a polyvinylidene difluoride membrane (Bio-Rad, L1620177). The membrane was soaked in 5% (w/v) skim milk for 2 h at room temperature. The membrane was incubated with primary antibodies against p-mTOR (Cell Signaling Technology, 5536, 1:1000), mTOR (Cell Signaling Technology, 2983, 1:1000), p-AKT (Cell Signaling Technology, 4060, 1:1000), AKT (Cell Signaling Technology, 4691, 1:1000), Atg7 (Cell Signaling Technology, 8558, 1:2000), p62 (Cell Signaling Technology, 5114, 1:2000), Atg5 (Cell Signaling Technology, 8540, 1:2000), LC3 (Cell Signaling Technology, 3868, 1:2000), APP (Cell Signaling Technology, 2452, 1:1000), BACE (Cell Signaling Technology, 5606, 1:1000) and ACTB (Cell Signaling Technology, 4970, 1:2500) overnight at 4°C. The membranes were washed three times in TBST (Tris-buffered saline with Tween 20 (Sigma, P1379, 1:1000)) for 5 min each, followed by incubation with HRP-conjugated anti-mouse (KPL, 5110-0011, 1:5000) or anti-rabbit (KPL, 5110-0010, 1:5000) antibody for 1 h at room temperature. The epitopes were visualized by using an ECL Western blot detection kit (FD BioScience, FD8030). Image J (National Institutes of Health, Bethesda, Maryland, USA) was used for the densitometry analysis. Western blotting of ACTB was used as a loading control to determine the densitometry of Atg7, Atg5, p62, p-AKT, AKT, p-mTOR, mTOR, APP and BACE. The LC3II densitometric signal was determined as the ratio of LC3II to LC3I before normalization to ACTB.

### Isolation of mouse microglia and analysis of microglial phagocytosis of Aβ

APP/PS1 mice (8-months-old) treated with IFN-γ or not were injected i.p. with Methoxy-X04 (10 mg/kg; Tocris, 4920, UK). After 3 h of Methoxy-X04 injection, the mice were deeply anesthetized and perfused with ice-cold saline. The isolated forebrain was cut into pieces and dissociated mechanically and enzymatically by using collagenase IV. 30% Percoll (Sigma-Aldrich, USA) was used to remove myelin and then labeled with AF488-conjugated mouse TMEM119 (1:100 dilution) antibodies. The data were analyzed by using BD FACSAria IIIu flow cytometer. The result microglia suspensions were used for the extraction of RNA by TRIzol.

### RNA of microglia extraction

For total RNA of microglia extraction, we used an optimized TRIzol protocol. Due to the high viscosity of the tissue and cell samples, the direct mRNA extraction could not be successfully accomplished, and RNA extraction was always performed using TRIzol. Either fresh or frozen tissues were homogenized with micropestles in 1 ml TRIzol. Chloroform extraction was performed using 0.2 ml, vortexed for 15 s and stew for 2–3 min at room temperature. Total RNA was then precipitated and pelleted using a 15 min centrifugation (12000 g) at 4 °C, followed by precipitation in 0.5 ml of isopropanol, vortexed for 15 s and stew for 10 min at room temperature. Then washed in 75% ethanol with a 5 min centrifugation (7500 g) at 4 °C and redissolved in 20 μl RNase-free water. The whole extraction protocol was developed on ice to avoid RNA degradation.

### Droplet digital polymerase chain reaction (ddPCR)

In our study, the QX200™ Droplet Digital™ PCR System (Bio-Rad, Hercules, CA, USA) was used. The ddPCR reaction mixtures contained: cDNA sample, 200 nM of primer, 1×x ddPCR™ Supermix for Probes (Bio-Rad, Pleasanton, CA, USA). Droplets were generated by using the QX200™ Droplet Generator (DG). A DG8™ cartridge holder and gasket with 20 μl of PCR reaction mixture and 70 μl of Droplet Generation Oil/well were used. In all, 40 μl of the generated droplets were transferred to ddPCR™ 96-well-plate. The plate was then heat-sealed using a PX1™ PCR plate sealer and a pierceable foil seal. The PCR thermocycling protocol was: initial denaturation at 95 °C for 5 min, 40 cycles of denaturation at 95 °C for 30 s, annealing at 60 °C for 1 min and extension for 1 min, followed by a final last incubation at 90 °C for 5 min and storage at 4 °C. The ddPCR™ 96-well-plate was placed into the QX200™ Droplet Reader. PCR droplets of each sample were analyzed and fluorescent signals of each droplet were quantified. All of the data was analysed by Quantalife (Bio-Rad, Hercules, CA, USA). The primer pairs: Atg7, 5′-TTGAGCGGCGACAGCATTAGG-3′ (forward) and 5′-CATGGCAGGAAAGCAGTGTGG-3′ (reverse); p62, 5′-GGCCCTGCTCAGTCTCTGAC-3′ (forward) and 5′-CCGGGGATCAGCCTCTGTAGAT-3′ (reverse); Atg5, 5′-ACACCCCTGAAATGAGTTTTCCAGA-3′ (forward) and 5′-CATCCAGAGCTGCTTGTGGTCT-3′ (reverse); LC3, 5′-TCAGATCGTCTGGCTCGGGA-3′ (forward) and 5′-GCGGCAGGAGAACCTACTGG-3′ (reverse).

### Statistical analysis

Statistical analyses were performed by using GraphPad Prism version 5.01 (GraphPad 5.01). Two-way ANOVA with repeated measure was used for the escape latency analysis of MWM. Other comparisons between two or more groups were performed with Student’s *t*-test (two-sided) or one-way ANOVA and followed by Bonferroni post hoc comparisons. Homogeneity test of variances and normal distribution were determined before each test. Data were expressed as the mean ± standard error of the mean (SEM) and *p* < 0.05 considered statistically significant.

## Supplementary information

Figure S1

Figure S2

Figure S3

Figure S4

Figure S5

Figure S6

Figure S7

Figure S8

Figure S9

Video.S1

Video.S2

Supplementary materials
