# Supplementary figures and images for: Intraperitoneal injection of IFN-γ restores microglial autophagy, promotes amyloid-β clearance and improves cognition in APP/PS1 mice
Source: Cell Death Dis. 2020 Jun 8;11(6):440. doi: 10.1038/s41419-020-2644-4 (PMC7280212; doi:10.1038/s41419-020-2644-4)

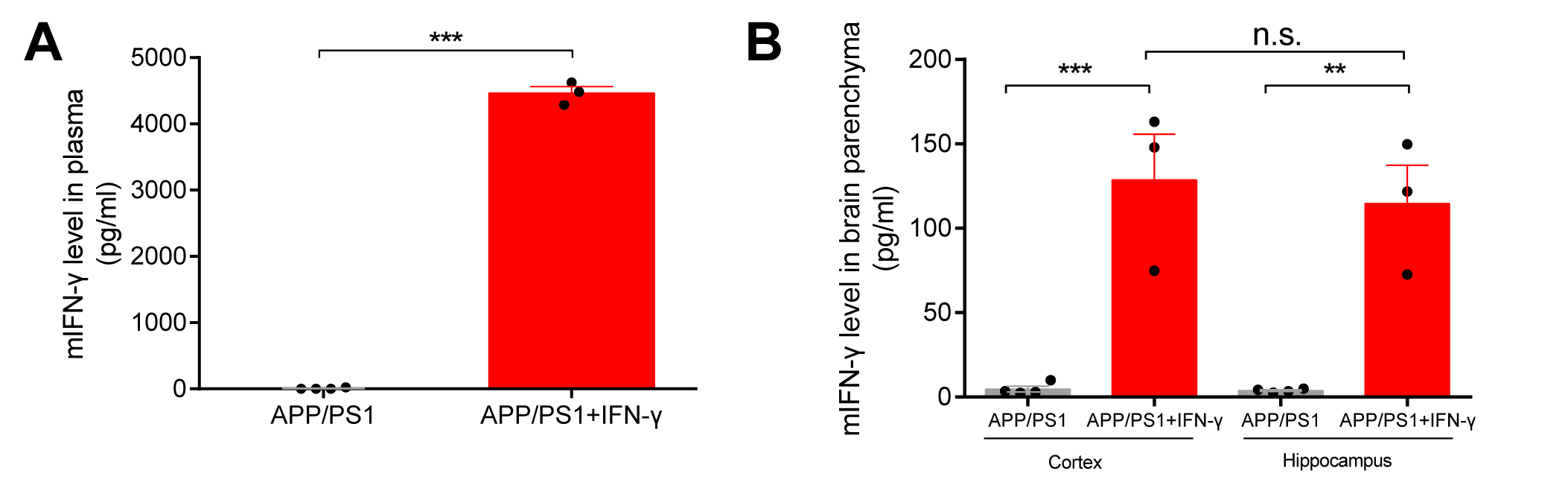

Supplement: Supplementary file 1 — Figure S1 [file 41419_2020_2644_MOESM1_ESM.tif]

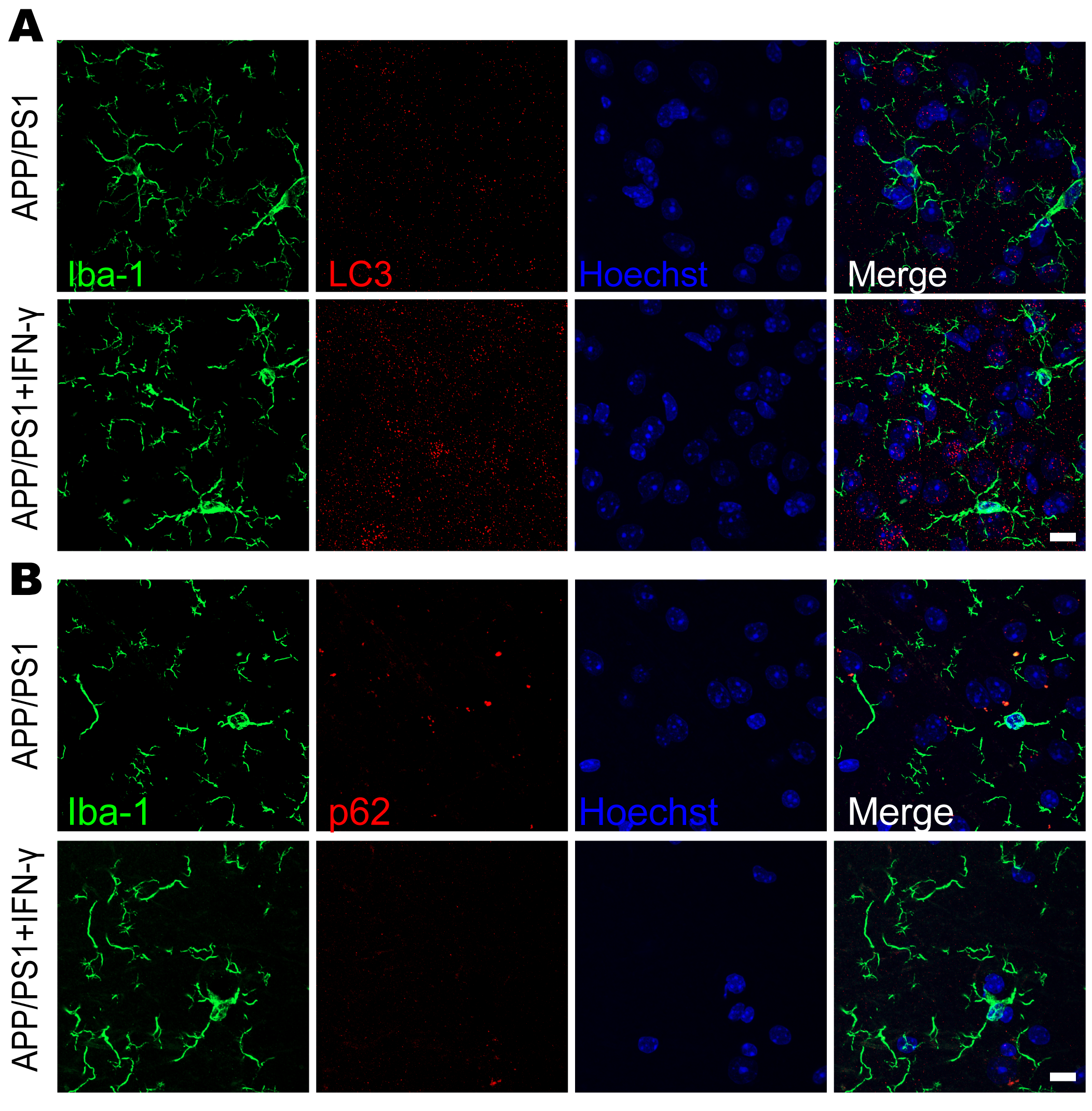

Supplement: Supplementary file 2 — Figure S2 [file 41419_2020_2644_MOESM2_ESM.tif]

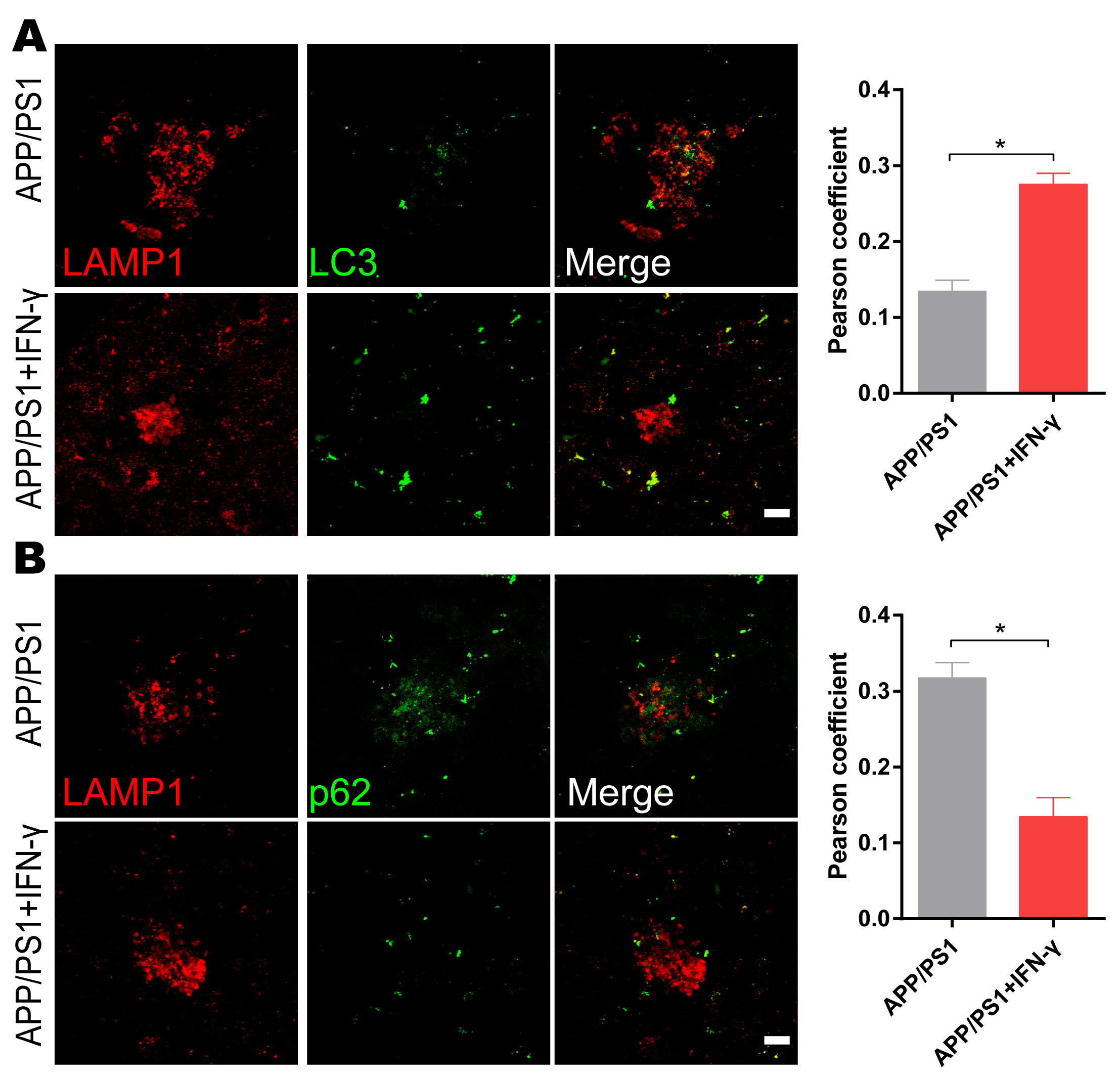

Supplement: Supplementary file 3 — Figure S3 [file 41419_2020_2644_MOESM3_ESM.tif]

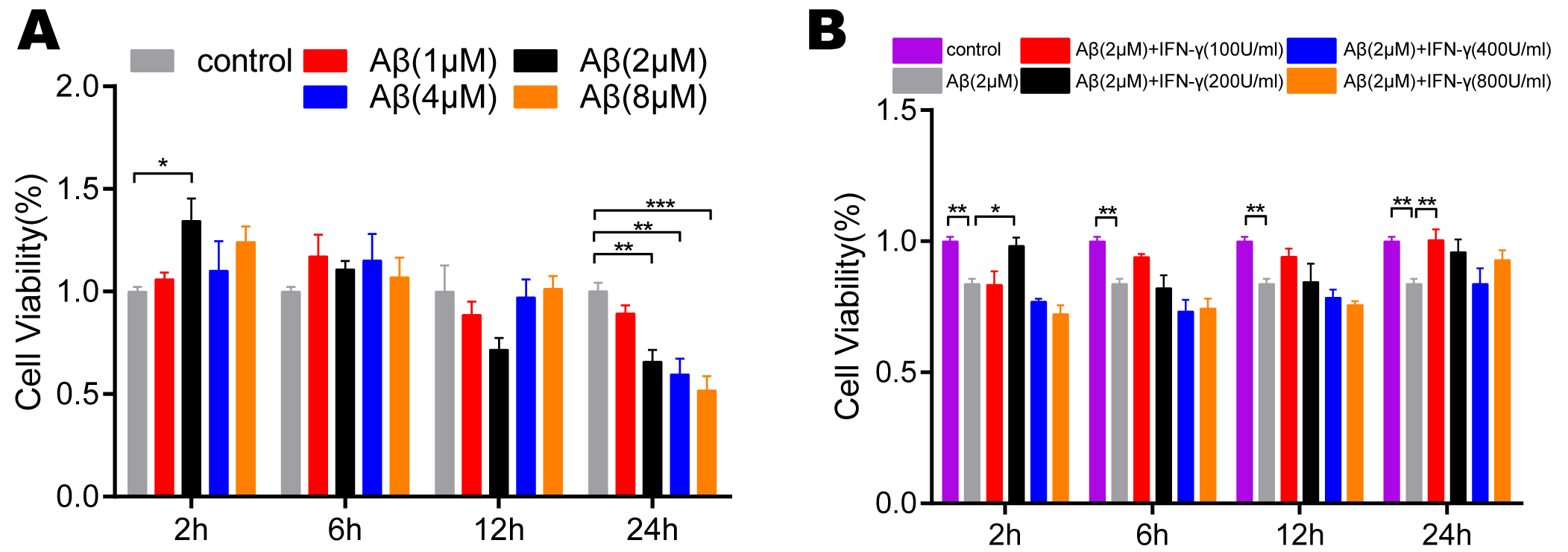

Supplement: Supplementary file 4 — Figure S4 [file 41419_2020_2644_MOESM4_ESM.tif]

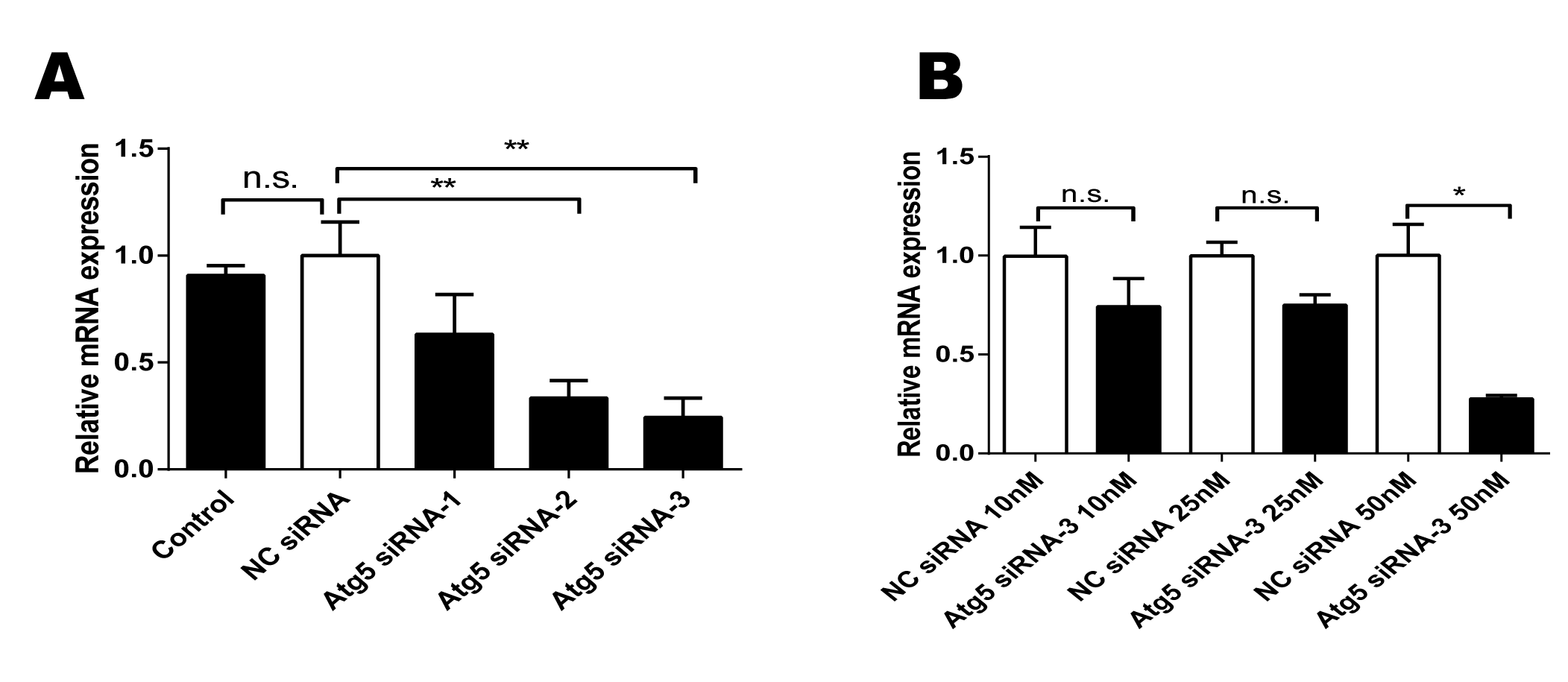

Supplement: Supplementary file 5 — Figure S5 [file 41419_2020_2644_MOESM5_ESM.tif]

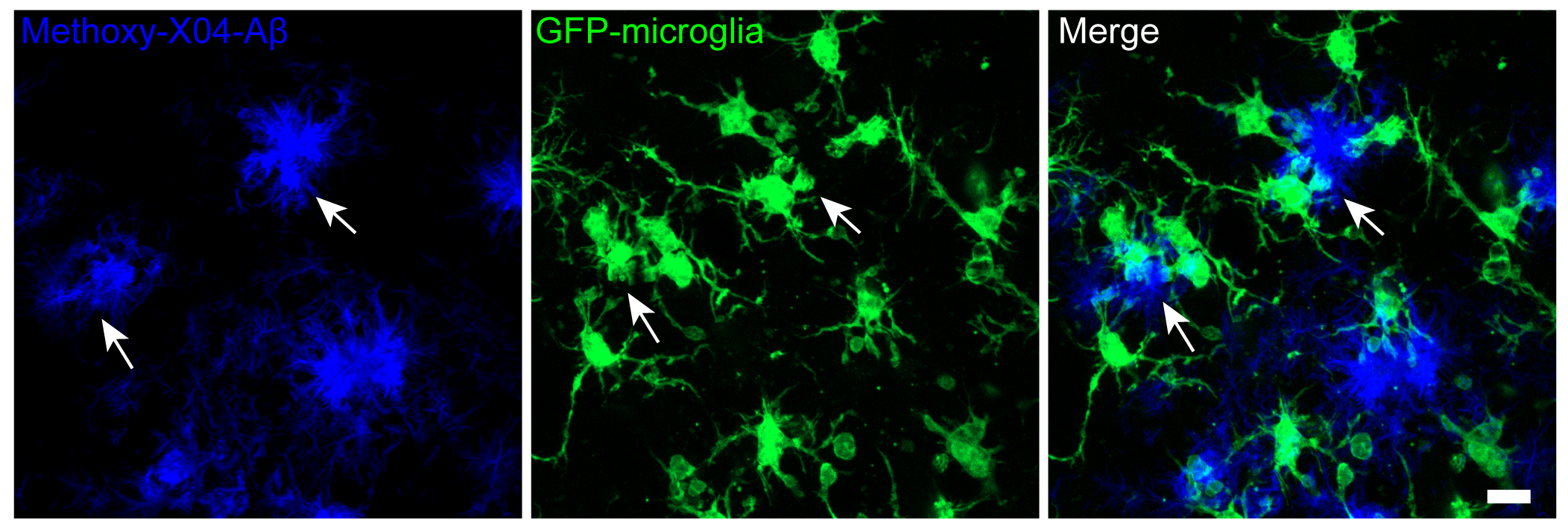

Supplement: Supplementary file 6 — Figure S6 [file 41419_2020_2644_MOESM6_ESM.tif]

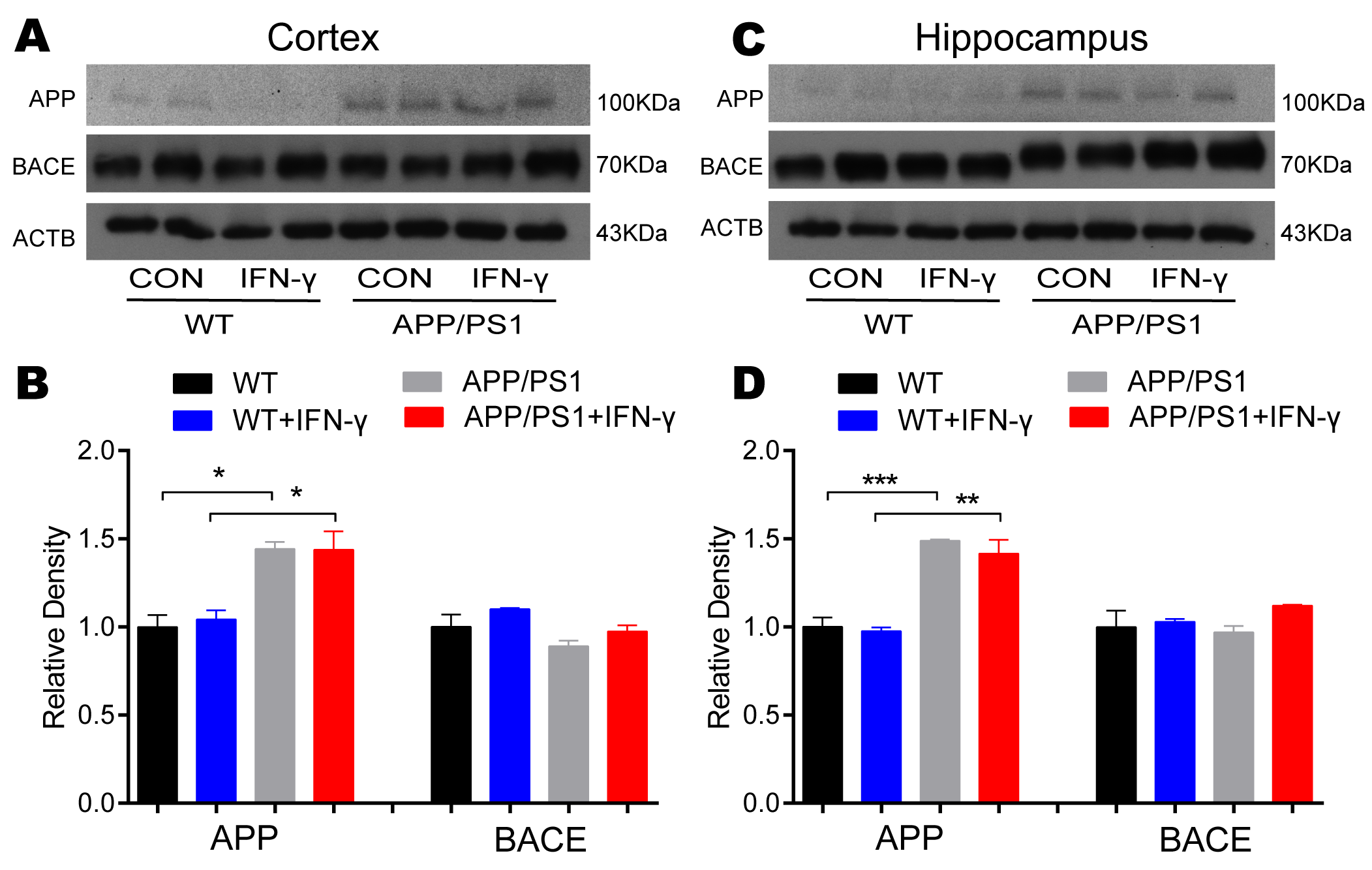

Supplement: Supplementary file 7 — Figure S7 [file 41419_2020_2644_MOESM7_ESM.tif]

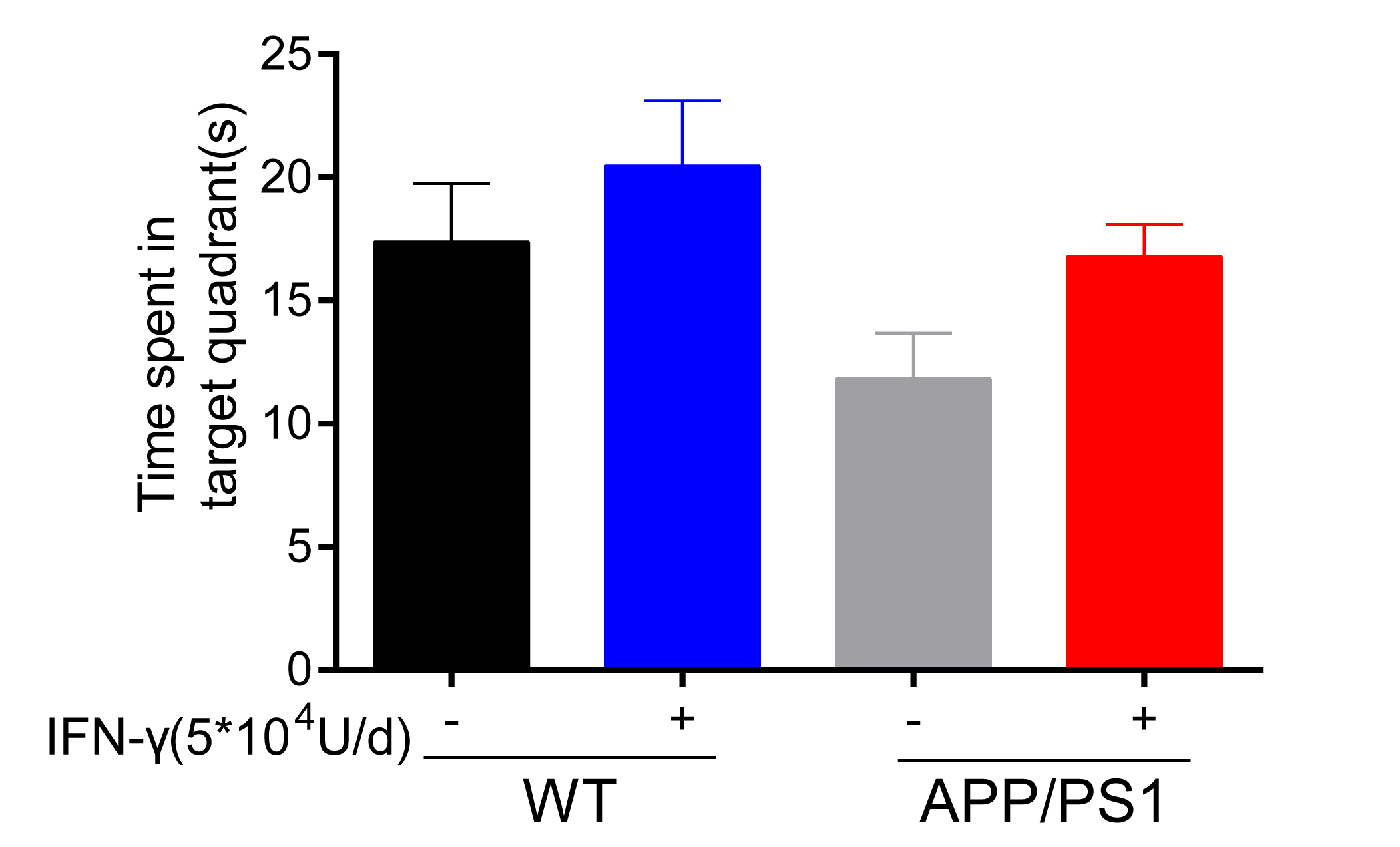

Supplement: Supplementary file 8 — Figure S8 [file 41419_2020_2644_MOESM8_ESM.tif]

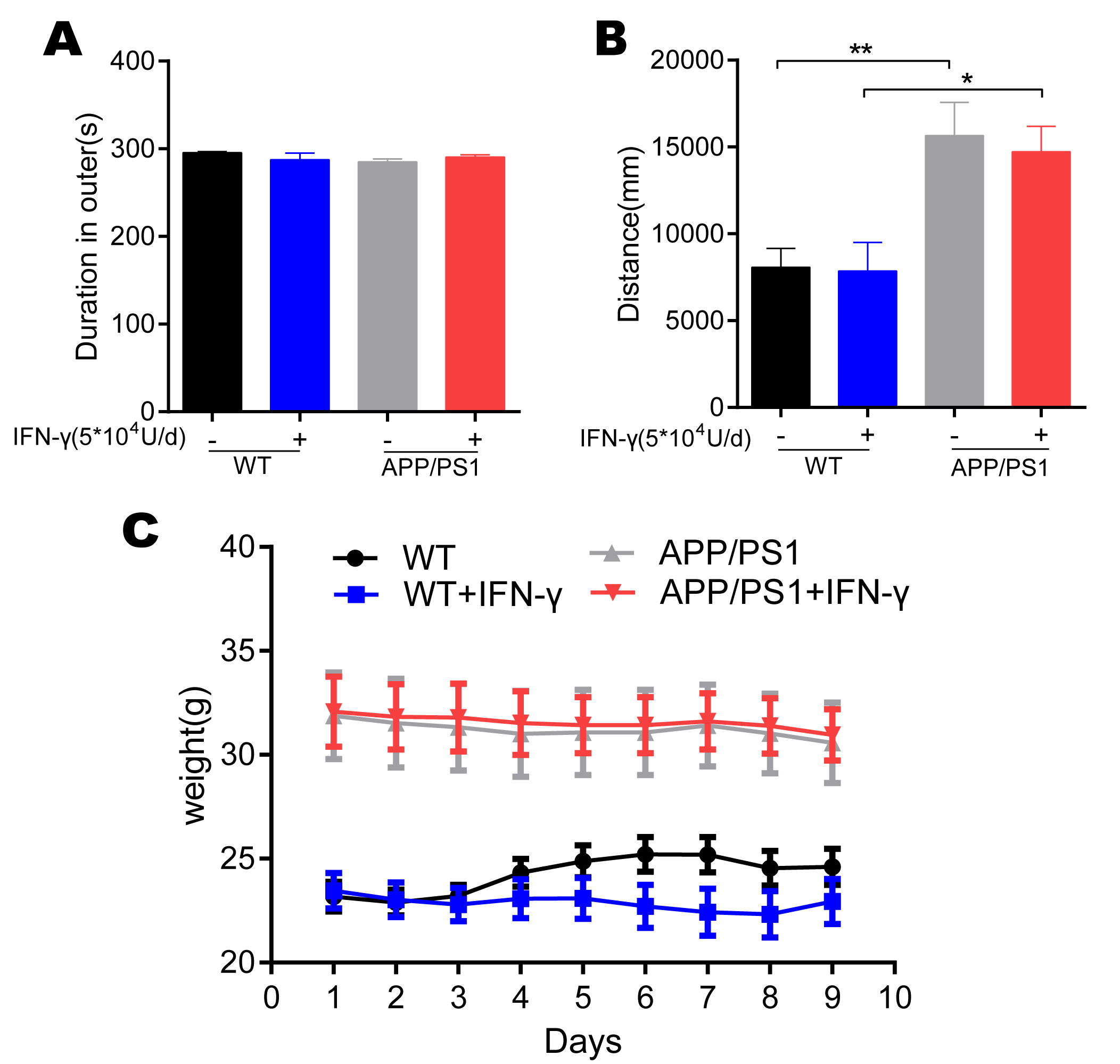

Supplement: Supplementary file 9 — Figure S9 [file 41419_2020_2644_MOESM9_ESM.tif]
