# Supplementary material for: Intraperitoneal injection of IFN-γ restores microglial autophagy, promotes amyloid-β clearance and improves cognition in APP/PS1 mice
Source: Cell Death Dis. 2020 Jun 8;11(6):440. doi: 10.1038/s41419-020-2644-4 (PMC7280212; doi:10.1038/s41419-020-2644-4)
Supplement: Supplementary file 12 — Supplementary materials [file 41419_2020_2644_MOESM12_ESM.docx]

**Supplementary materials**

Fig. S1 **Increased IFN-γ levels in the brain tissues of APP/PS1 mice following intraperitoneal (i.p.) administration of IFN-γ.** **a, b** APP/PS1 mice (8 months old) were i.p. injected with murine IFN-γ (5×10^4^ U) for 30 min. IFN-γ levels in the plasma (**a**), cortex and hippocampus (**b**) were measured by ELISA kit. Data are the mean ± SEM, APP/PS1: n = 4/group, APP/PS1+IFN-γ: n = 3/group. (**P* < 0.05, ****P* < 0.001, Student’s *t*-test).

Fig. S2 **IFN-γ induces autophagy in the plaque-free areas. a** Representative images of staining with anti-LC3 (red), anti-Iba-1 (green) antibodies and Hoechst stain (blue) in APP/PS1 mice treated with IFN-γ or not. **b** Representative images of staining with anti-p62 (red), anti-Iba-1 (green) antibodies and Hoechst stain (blue) in APP/PS1 mice treated with IFN-γ or not. Scale bar = 10 μm.

Fig. S3 **IFN-γ increases the expression of LAMP1.** **a** Representative confocal images and quantitative analyses. Sections were stained with LAMP1 (red) antibodies to label lysosomes and LC3 (green) antibodies. The pearson coefficient was significantly increased in APP/PS1 mice treated with IFN-γ. **b** Representative confocal images and quantitative analyses. Sections were stained with LAMP1 (red) antibodies to label lysosomes and p62 (green) antibodies. The pearson coefficient was significantly decreased in APP/PS1 mice treated with IFN-γ. Scale bar = 10 μm. **P* < 0.05. Data were analyzed by Student’s *t*-test and are presented as the mean ± SEM. (n = 3/group.)

Fig. S4 **IFN-γ protected BV2 cell viability from Aβ-induced cytotoxicity.** **a** BV2 cells (10^4^ cells/well) were incubated with different Aβ concentrations (1 μM, 2 μM, 4 μM, and 8 μΜ) for 2 h, 6 h, 12 h or 24 h in 96-well plates. BV2 cell viability was measured by a CCK-8 assay. The selected effective concentration for assays of Aβ-induced cytotoxicity was 2 μM over 24 h. **b** BV2 cells (10^4^ cells/well) were incubated in 96-well plates in 2 μM Aβ for 24 h and then incubated with various IFN-γ concentrations (100 U/ml, 200 U/ml, 400 U/ml, and 800 U/ml) for 2 h, 6 h, 12 h or 24 h. The cell viability of BV2 cells was measured by a CCK-8 assay. The selected effective concentration of IFN-γ-resistant Aβ was 200 U/ml for 2 h. **P* < 0.05, ***P* < 0.01, ****P* < 0.001, one-way ANOVA and Bonferroni *post hoc* test. Data are representative of 3 independent experiments with similar results and shown as the mean ± SEM.

Fig. S5 **The knockdown efficiency of the Atg5 gene by the siRNAs in BV2 cells.** **a** BV2 cells were cultured in DMEM medium with 10% FBS and transfected with 3 siRNAs (50 nM) against the Atg5 gene. Atg5 siRNA-3 was the most efficient and was selected for the following experiments. **b** The most effective concentration was 50 nM. Each treatment was performed in a 6-well plate. NC siRNA, negative control siRNA. **P* < 0.05, ***P* < 0.01, Student’s *t* test. Bars represent the mean ± SEM. These results were based on 3 independent experiments.

Fig. S6 **CX3CR1-GFP positive cells interact with the endogenous Aβ labeled with Methoxy-X04.** Methoxy-X04 was injected into the APP/PS1; Cx3cr1-GFP^+/-^ mice through tail vein (50 μl, 5 mg/kg) and representative fluorescent image of Aβ (blue) and CX3CR1-GFP^+/-^ cells (green) in the APP/PS1; Cx3cr1-GFP^+/-^ mice. CX3CR1-GFP positive cells interact with the endogenous Aβ labeled with Methoxy-X04, as indicated by the white arrows. Scale bar = 10 μm

Fig. S7 **IFN-γ had no effect on the APP processing in APP/PS1 mice.** **a, c** Representative immunoblot of APP and BACE immunoreactivity in the cortex (**a**) and hippocampus (**c**) of APP/PS1 mice treated with IFN-γ. **b, d** Intensity analysis of APP and BACE bands normalized to ACTB showed no significant changes in APP and BACE levels in the cortex (**b**) and hippocampus (**d**) of APP/PS1 mice treated with IFN-γ. **P* < 0.05, ***P* < 0.01, ****P* < 0.001, one-way ANOVA and Bonferroni *post hoc* test. The results are all shown as the mean ± SEM. (n = 3/group)

Fig. S8 **The time spent in the target quadrant was not affected in APP/PS mice treated IFN-γ or not.** One-way ANOVA and Bonferroni *post hoc* test. The results are all shown as the mean ± SEM. (n = 9/group)

Fig. S9 **Physical activity and body weight were not affected by IFN-γ treatment.** **a** The duration of the outer time of all groups was similar. **b** IFN-γ did not affect the distance traveled by mice. **c** IFN-γ had no effect on the WT and APP/PS1 mice weight. **P* < 0.05, ***P* < 0.01, one-way ANOVA and Bonferroni *post hoc* test. The results are all shown as the mean ± SEM. n = 9/group.

Video. S1 **CX3CR1-GFP positive cells surrounding the exogenous Aβ labeled with Rhodamine.** Rhodamine-Aβ was stereotactically injected into the lateral ventricles of CX3CR1-GFP^+/-^ mice and recorded using a two-photon microscope. Microglia (green) surrounded Rhodamine-Aβ (red), as indicated by the white arrows. Scale bar = 20 μm

Video. S2 **CX3CR1-GFP positive cells surrounding the endogenous Aβ labeled with Methoxy-X04.** Methoxy-X04 was injected into the APP/PS1; Cx3cr1-GFP^+/-^ mice through tail vein (50 μl, 5 mg/kg) and recorded using a two-photon microscope. CX3CR1-GFP^+/-^ cells surrounded Aβ-labeled with Methoxy-X04 (blue), as indicated by the white arrows. Scale bar = 20 μm
